# Review of Optical Thermometry Techniques for Flows at the Microscale towards Their Applicability to Gas Microflows

**DOI:** 10.3390/mi13111819

**Published:** 2022-10-25

**Authors:** Stéphane Colin, José M. Fernández, Christine Barrot, Lucien Baldas, Slaven Bajić, Marcos Rojas-Cárdenas

**Affiliations:** 1 Institut Clément Ader (ICA), Université de Toulouse, CNRS-INSA-ISAE-Mines Albi-UPS, 31400 Toulouse, France; 2 Fédération de recherche FERMAT, CNRS, 31400 Toulouse, France; 3 Laboratory of Molecular Fluid Dynamics, Instituto de Estructura de la Materia IEM-CSIC, 28006 Madrid, Spain

**Keywords:** thermometry, gas microflow, microsystem, microfluidics, temperature, rarefaction

## Abstract

Thermometry techniques have been widely developed during the last decades to analyze thermal properties of various fluid flows. Following the increasing interest for microfluidic applications, most of these techniques have been adapted to the microscale and some new experimental approaches have emerged. In the last years, the need for a detailed experimental analysis of gaseous microflows has drastically grown due to a variety of exciting new applications. Unfortunately, thermometry is not yet well developed for analyzing gas flows at the microscale. Thus, the present review aims at analyzing the main currently available thermometry techniques adapted to microflows. Following a rapid presentation and classification of these techniques, the review is focused on optical techniques, which are the most suited for application at microscale. Their presentation is followed by a discussion about their applicability to gas microflows, especially in confined conditions, and the current challenges to be overcome are presented. A special place is dedicated to Raman and molecular tagging thermometry techniques due to their high potential and low intrusiveness.

## 1. Introduction

Following recent advances in the fabrication of microelectromechanical systems (MEMS), new and exciting applications for microfluidics have emerged. A large amount of research, both on the theoretical and experimental fronts, has been carried out in microfluidics during the last three decades [1,2,3,4,5,6]. The applications of this field concern rich and varied domains spanning from biology to spacecraft research. More specifically, regarding gas microflows, several practical microsystems have been developed, such as micronozzles [7,8], micro heat exchangers [9,10], micro actuators [11], micro thrusters [12,13], micro gas chromatographs [14,15], gas sensors and separators [16,17,18], and vacuum micropumps [19]. Most of these applications require a control of the heat transfer and temperature distributions inside the gas flow. In addition, the determination of convective heat transfer coefficients for flows in microchannels is directly related to the knowledge of temperature distribution in these channels [20]. The knowledge and mastering of temperature distributions at an experimental level is not only of engineering applications interest, but it is also of fundamental importance for validating the theoretical models which are commonly used to predict fluid flow and heat transfer at the microscale [21,22,23].

With the arrival of microfluidics, the first experimental approach towards the measurement of temperature distributions at the microscale was to scale down the existing macroscale measurement techniques [20]. However, many of these techniques were not viable at microscale, mainly due to their high intrusiveness leading to flow perturbations. This has led researchers to investigate and develop new experimental techniques, making the field of microscale thermometry an active area of research currently. Even if reliable experimental data are now available for both hydrodynamics and heat transfer in liquid microflows, this is unfortunately not yet the case for gas microflows.

### 1.1. Fundamental Interest for Experimental Thermometry in Gas Microflows

**Figure 1:**
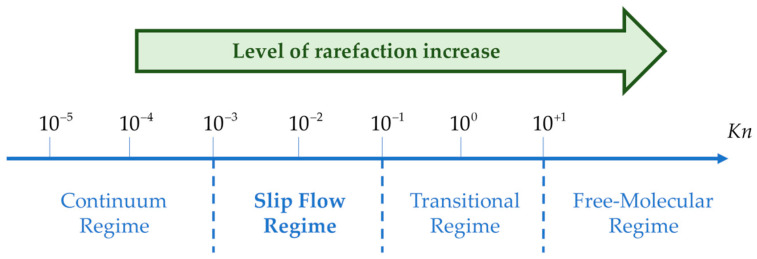
Classification of flow regimes based on Knudsen number.

In gas microflows, the Knudsen number Kn=l/L, defined as the ratio of the molecular mean free path l to a characteristic dimension *L* of the system, is a dimensionless number quantifying the flow rarefaction [24]. Depending upon the value of the Knudsen number, the flow can be classified into four main rarefaction regimes: continuum, slip flow, transition and free molecular regimes, as shown in Figure 1.

**Figure 2:**
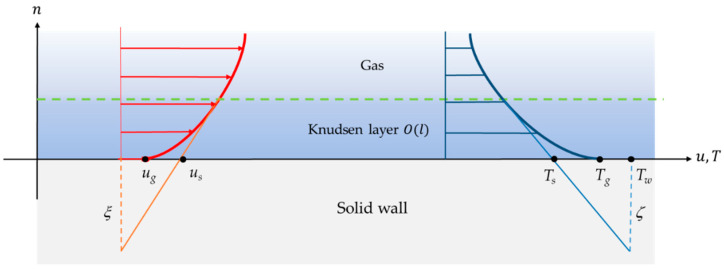
Schematic depicting velocity slip and temperature jump at the wall.

In microsystems with gas flows, a Knudsen number in the range 10^−3^–10^−1^ is frequently encountered, and this corresponds to the moderately rarefied slip flow regime. In this case, the flow exhibits local thermodynamic disequilibrium in the near-wall region, in the so-called Knudsen layer (Figure 2). This local disequilibrium introduces a discontinuity of velocity and temperature between the gas and the wall. Modeling the gas flow using a continuum approach is, however, still possible, provided classical boundary conditions are replaced with specific boundary conditions that account for a velocity slip and a temperature jump at the wall. Theoretical investigations have revealed the strong influence of the velocity slip and temperature jump at the wall on the heat transfer properties in rarefied flows [23].

Figure 2 shows a representation of the velocity slip and temperature jump experienced by the gas in contact with a non-moving wall. In this figure, ξ is the velocity slip length, $u_{g}$ is the velocity of the gas at the wall, and $u_{s}$ is the velocity corresponding to a linear extrapolation of the velocity profile out of the Knudsen layer. Similarly, ζ is the temperature jump distance, $T_{w}$ is the temperature of the wall, $T_{g}$ is the temperature of the gas at the wall, and $T_{s}$ is the temperature corresponding to a linear extrapolation of the temperature profile out of the Knudsen layer:

(1) $$T_{s}-T_{w}=\zeta {\left(\frac{\partial T}{\partial n}\right)}_{w}$$

Equation (1) provides a general description of the expected temperature jump at the wall, where ζ is directly proportional to l and n represents the direction normal to the wall oriented towards the gas. Several models of ζ are proposed in the literature [25].

Experimental data should be very useful for directly measuring ζ and also obtaining information on the temperature distribution within the Knudsen layer, in order to discuss the accuracy of the models and to better understand the mechanisms of gas/surface interaction in local disequilibrium. This would enhance the development of new micromechanical systems with optimized properties. Available experimental data on confined rarefied gas flows, however, are limited. More specifically, very few local measurements of velocity and temperature have been published [26], especially concerning near-wall regions.

The present review on microscale thermometry in fluids discusses the currently available techniques for the measurement of temperature in microflows. Following a rapid presentation and classification of the various thermometry techniques, the review is focused on optical techniques. The optical thermometry techniques are the most appropriate for application at microscale, and their presentation is followed by a discussion about their applicability and effectiveness in the case of gases.

### 1.2. Classification of Temperature Measurement Techniques

Temperature is a quantity directly linked to the thermal energy of a medium, which, for gases, can be quantified in terms of the microscopic energy of the molecules. This energy is mainly due to translational, rotational and vibrational motions of the molecules, plus, in some cases, a non-negligible contribution of the electronic motion. The actual thermal energy contributions depend on the number of degrees of freedom of the gas molecules: the energy of a monatomic gas originates solely from the three degrees of freedom of its translational movement, while a diatomic molecule also has two degrees of freedom of rotational motion plus one degree of freedom of vibrational motion. More complex polyatomic molecules have additional degrees of freedom. At very low temperatures, the degrees of freedom are restrained, and the internal molecular energy is essentially limited to its translational contribution. At temperatures from low to moderate, the rotational motions become more active, while the vibrational modes are usually activated above room temperature. At very high temperatures the electronic motion can also contribute significantly to the molecular internal energy.

It is worth noting that each of these contributions can be associated to a particular temperature, which are all equal to the thermodynamic temperature when the gas is at equilibrium. However, gases in microflows are often out of equilibrium, and then one may have to speak about different rotational, vibrational and translational temperatures. This differentiation also manifests macroscopically, since each internal motion relaxes with its particular characteristic time towards equilibrium [27].

In all cases, it is important to note that the estimation of temperature can only be based on observable temperature dependent effects [28].

The most general way of classifying thermometry techniques is based on the intrusiveness of the sensing element [29,30,31,32], and contact, non-contact or semi-contact-based techniques are distinguished. Contact measurement thermometers (e.g., thermocouples and resistance temperature detectors) are of common use, but more sophisticated systems, such as atomic force microscopy temperature probes, have also been developed. Contactless techniques (e.g., interferometry, see Section 5) can ensure completely non-intrusive temperature measurements, while certain techniques can be classified as semi-intrusive techniques since the tracers which are seeded within the carrier fluid can be considered as intrusive to a certain extent (e.g., molecular tagging, see Section 7).

Another system of classification is based on the calibration procedure used for the technique employed. In primary systems, well-established equations of state are used for directly relating the measured signal to the absolute temperature. In secondary systems, the link between temperature and measured quantity is not straightforward and it is necessary that the measured signal has a reference to a precise temperature (within experimental uncertainties). In some instances, however, calibration may pose considerable difficulties.

**Figure 3:**
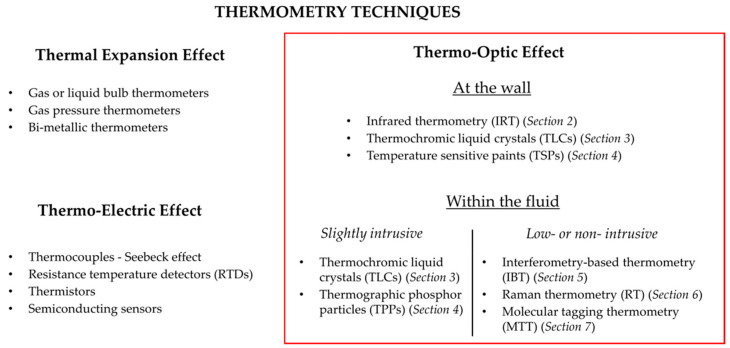
Classification of various temperature measurement techniques according to the underlying physical principle exploited.

Lastly, one can also classify the thermography techniques according to the underlying physical principle exploited. The techniques are based on thermal expansion, the thermo-electric effect or the thermo-optic effect. This kind of classification has been chosen for the purpose of this review (Figure 3).

Techniques based on thermal expansion are hardly applicable to microscale, as they require using a non-negligible additional volume of gas, liquid or solid. For this reason, they are not detailed in this paper. Micro-sensors exploiting a thermo-electric effect are briefly described in Section 1.3, but the review is focused on less intrusive techniques based on thermo-optic effects, which are analyzed in more detail in Section 2, Section 3, Section 4, Section 5, Section 6 and Section 7, as listed in Figure 3.

### 1.3. Conventional and Contact-Based Thermometry Adapted to Microscale

This section briefly presents the conventional contact-based temperature measurement techniques adapted to microscale. These techniques were conventionally used for measuring temperature at macroscale, and with the development of modern fabrication techniques, sensors have been miniaturized for microscale temperature measurement.

Thermocouples are based on the Seebeck effect. A thermocouple is essentially formed by joining two dissimilar metals with two junctions. By imposing a temperature difference between these two junctions, one of them being at a reference temperature, an intrinsic Seebeck voltage is produced. The relationship between temperature and voltage is almost linear, resulting in simple calibration curves. With the advances in microfabrication, it has become common to fabricate micro- or nano-thermocouples using different manufacturing methods [33]. For instance, Zhang et al. [34] have designed, fabricated and characterized metal embedded thin film thermocouples considering various film thicknesses and junction sizes. Thiery et al. [35] have carried out temperature profile measurements of near-field optical microscopy fiber tips by means of sub-micrometric thermocouples. Recently, researchers have been able to fabricate gold-nickel (Au-Ni) thermocouples with 2.5-μm-wide electrodes on a 30-nm-thick silicon nitride (Si_3_N_4_) membrane using electron beam lithography [36]. The main disadvantages of thermocouples are their short lifetime and low robustness. Sometimes they are very sensitive to the presence of external sources of heat and may be prone to corrosion. During the operation of a thermocouple, the main sources of error are noise and drift. In addition, their temperature sensitivity is generally quite low, of the order of 50 µV K^−1^ or less, and their accuracy remains limited typically to ±0.5 K [37]. In the case of micro-thermocouples, there are fabrication difficulties in forming a junction using thin wires and thereby making a useful and reliable device. On the other hand, the low volume of micro-thermocouples significantly reduces their thermal inertia, allowing high frequency operation, with a response time of a few tens of ns [38]. In conclusion, thermocouples are cost-effective and can measure different ranges of temperature as a function of the thermocouple type, i.e., according to the choice of the couple of metals.

Other contact-based techniques, such as micro resistance temperature detectors (µRTDs) [39,40,41], thermistors [42,43,44,45] and other kinds of semiconducting sensors [46,47,48,49] are employed in various studies described in the literature.

RTDs exploit the increase of electrical resistance of metals with temperature. The use of platinum allows a linear relationship between resistance and temperature, coupled with a chemical inertness. Compared with thermocouples, RTDs present a higher stability and higher sensitivity, of the order of 350 µV K^−1^, as well as a higher accuracy, typically in the range ±0.05–0.1 K [37]. Moreover, their response time is higher.

Thermistors are similar to RTDs in their operating principle, but they are made in semiconductor materials or transition metal oxides and their resistance decreases as the temperature increases. Their nonlinear relationship between resistance and temperature practically limits the range of temperature they can cover. Their advantages are a very high sensitivity, of the order of 10,000 µV K^−1^, and accuracy, typically ±0.02–0.05 K [37].

All of the previously contact-based sensors are adequate for both gas and liquid thermometry, but they allow a measurement of temperature only at discrete locations, without any direct information about continuous spatial distribution. In addition, these sensors do not allow temperature measurement within the bulk flow without a strong perturbation of the flow dynamics and heat transfer. Therefore, though the above-mentioned methods may be cost-effective and easy to use, they are mainly limited to local measurements at the walls. This limitation has led to the development of further sophisticated optical-based techniques.

### 1.4. Optical-Based Techniques

In this review, various optical-based techniques applied to the temperature measurement of fluids at microscale are presented and analyzed. Optical techniques have been widely employed at microscale due to their unique characteristic of non- or low-intrusiveness and their ability to provide superior spatial and temporal resolution [50]. Each following section devoted to a specific technique is introduced by a brief description of its physical principle, followed by example applications to liquid microflows and to gas flows. A discussion on the applicability of the technique to the thermometry in gas microflows then concludes the section.

Different techniques could be coupled to provide temperature data both at the wall and within the gas. For each technique, a figure summarizing the pros and cons is presented. Some optical techniques are not appropriate for gases or microfluidic applications and they have been excluded from this review. For example, the inherent invasive nature of the laser-induced breakdown thermometry technique could significantly alter the temperature and density distributions of the flow by forming plasma [51].

## 2. Infrared Based Techniques

### 2.1. Principle of Infrared Imaging

A body at a temperature around ambient values emits electromagnetic radiation in the infrared (IR) band of the electromagnetic spectrum, i.e., with a wavelength from 700 nm to 1 mm. Based on this phenomenon, infrared thermography (IRT) consists in transforming the energy radiated from the body into an electronic signal by means of a radiometer (the infrared sensor of the IR cameras). The signal is then converted into an image that maps the different infrared radiation levels represented in colors or grayscale.

Planck’s law describes the energy distribution from a blackbody at a given temperature as a function of the emission wavelength. However, some bodies may have a radiative behavior far from the blackbody one. By knowing the emissivity, that is the ratio of the radiation emitted by the body to the radiation emitted by the blackbody at the same temperature, it is possible to determine the temperature of a specific body. The precise knowledge of the emissivity of materials is then essential for temperature measurements using IRT, with the difficulty that the emissivity can be a function of both the wavelength and the direction of radiative emission. Also, great care must be taken to include additional parameters in the data analysis, such as reflections of thermal radiations from nearby sources, to ensure a reliable estimate of the temperature. A calibration process is necessary to consider the influence of all the surrounding elements on the energy detected by the sensor. As a rule of thumb, it can be argued that low reflectivity materials due to their high emissivity are better suited for IR imaging.

The performances of an infrared system are evaluated in terms of thermal sensitivity (for current devices it can be less than 20 mK), scan speed (that can be higher than 1600 Hz), image resolution (up to tens of thousands of pixels) and intensity resolution (up to 16-bits) [52]. It is an efficient tool for mapping the surface temperature of solids or liquids, for which radiation can be considered as a surface phenomenon, as the radiation emitted by the molecules that do not belong to a thin surface layer is directly absorbed by the body itself.

In the case of gases, radiation is really a volumetric phenomenon, as the medium is transparent to radiation emitted by all the gas molecules. Nonpolar gases, such as O_2_ or N_2_, do not emit radiation and are essentially transparent to incident thermal radiation. It is, however, different for polar gaseous molecules, such as CO_2_, H_2_O, NH_3_, or hydrocarbon gases, which emit and absorb over a wide temperature range [53]. In addition, gaseous radiation is complicated by the fact that, unlike radiation from a solid or a liquid, which presents a continuous emission spectrum, gaseous radiation is concentrated in specific wavelength intervals that are called bands. Each molecule at the gaseous state has its own radiative emission spectrum which requires the gathering of a complex database in order to associate infrared radiative emission to temperature [54]. For internal gas flows, it is generally more straightforward to measure the radiations of the walls and to deduce, with appropriate hypotheses, information on the temperature distribution within the gas.

Despite these difficulties, IRT has several advantages as it is non-intrusive and contactless. In the case of internal fluid flows, however, direct fluid IRT cannot be easily implemented, since most materials are not transparent to IR radiation. In addition, even specific materials partly transparent to some IR radiations emit their own radiation, which can lead to the detection of a wrong signal or to an image with a low signal over noise ratio.

### 2.2. Applications of IRT to Liquid Microflows

At microscale, the limitations are due to diffraction phenomena: the spatial resolution of a pixel cannot be lower than the wavelength of the IR signal (i.e., lower than about 5 µm). On the other hand, the acquisition frequency depends on the image size, the integration time and the type of data storage, and quite high frequencies of the order of some kHz can be obtained for images of limited size. Many studies on IR measurements in liquids microflows have been published, and some of them propose solutions to overcome spatial and temporal limits (see Section 2.2.1 and Section 2.2.2).

#### 2.2.1. Liquid Temperature Estimation through Measurement of the Wall Temperature

Hetsroni et al. [55] measured external surface temperatures in small channels (1070 µm in diameter) filled with water flowing at Reynolds numbers ranging from 10 to 400. The methodology they proposed allows to limit systematic errors caused by the radiations of the surrounding elements. Once calibrated, the radiometer used in this study gave a typical noise equivalent to a temperature difference of 0.07 K, which was lower than the sensitivity of the system. A detailed study leads to a value of standard uncertainty for the temperature wall of 0.29 K. In a similar way, Patil and Narayanan [56] employed the IR technique to measure the wall and near-wall temperatures of water flowing through a silicon microchannel 50 µm wide and 135 µm deep. They worked at a temperature of about 45 °C and were able to determine the local temperatures with an uncertainty of 0.60 and 1.33 K for Reynolds numbers of 297 and 251, respectively.

**Figure 4:**
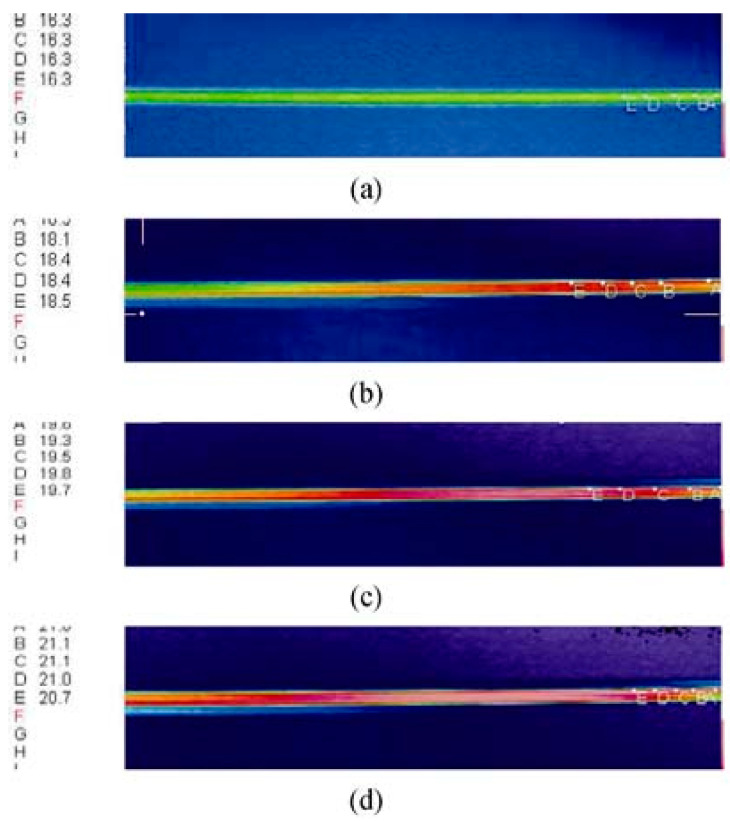
Surface temperature measured by Liu et al. [57] in a microtube of 44.2 µm inner diameter and 140 mm length at various Reynolds numbers. (**a**) Re=0; (**b**) Re=297.2; (**c**) Re=491.8; (**d**) Re=692. Temperatures for various locations in the channel are provided in °C in the left part of the image. Reprinted by permission from Ref. [57], Journal of Thermal Science, Copyright © 2022 Springer-Verlag.

Liu et al. [57] employed IR imaging techniques to study the effects of viscous dissipation in quartz glass microtubes with inner diameters of 19.9 µm and 44.2 µm. De-ionized water was the working fluid. Depending on the Reynolds number *Re* based on the microtube radius, the temperature difference between the inlet and outlet of the fluid varied from 1 to about 10 °C. The temperature inside the fluid was deduced from surface temperature measurements of the outer wall with corrections considering convection in the fluid and conduction in the microchannel wall. Figure 4 shows the surface temperature measurements at various *Re*. The uncertainties on IR measurement reported in this study were about 0.3 K.

Hetsroni et al. [58] combined the IR technique with high speed flow visualization to simultaneously measure the surface temperature and the flow pattern of two-phase flows in microchannels in order to explore the relationship between temperature surface, heat flux and bubble generation during boiling. The working fluids were air-water and steam-water flowing in a series of parallel triangular microchannels with hydraulic diameters of 103, 129 and 161 µm. The authors were able to map temperatures in the range of 50 to 120 °C with a radiometer sensitivity of 0.1 K by averaging them over a time interval of 0.04 s.

**Figure 5:**
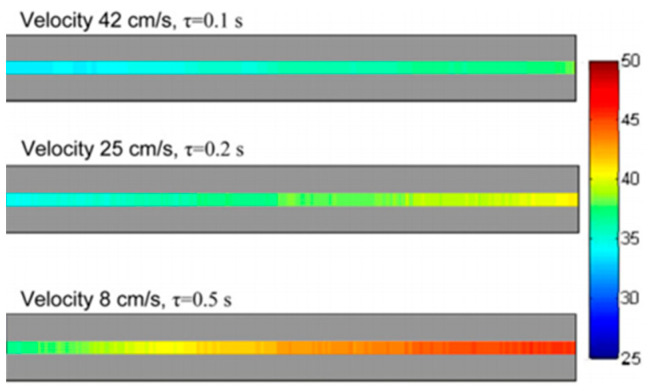
Temperature profiles in a microchannel reactor during the hydrolysis of tetraethoxysilane at different flow velocities and times, from Haber et al. [59]. The scale bar provides the correspondence between colors and temperatures in °C. Reprinted by permission from Ref. [59], Chemical Engineering Journal, Copyright © 2022 Elsevier.

Haber et al. [59] studied by IR imaging the fast and exothermic reaction of tetraethoxysilane hydrolysis by mapping temperature profiles in a micro-reactor made of two parallel rectangular microchannels (one for the reaction and one for cooling) in a Polyether ether ketone (PEEK) substrate. The cross section of the reaction channel had a depth of 100 µm and a width of 500 µm, and the PEEK wall had a thickness of 250 µm. Thanks to a calibration, they were able to estimate temperature profiles between 30 and 50 °C at different flow velocities and time intervals, as shown in Figure 5, by IR imaging of the PEEK wall external surface. The reported accuracy of their temperature measurements was about 1 K with a spatial resolution of 200 µm/pixel.

#### 2.2.2. Measurement of Liquid Surface Temperature

**Figure 6:**
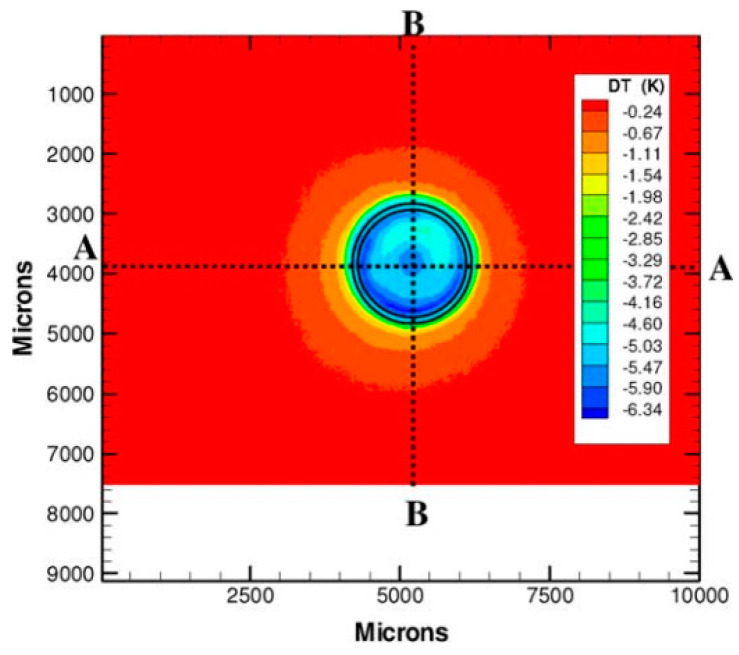
Temperature of the meniscus of methanol evaporating in a 1630 µm diameter capillary tube. IR image obtained after subtraction of the same image taken with an empty tube by Buffone and Sefiane [60]. Reprinted by permission from Ref. [60], Experimental Thermal and Fluid Science, Copyright © 2022 Elsevier.

IR techniques have also been widely used in evaporation studies. In some cases, IR measurements allow for the direct mapping of the temperature of the gas-liquid interface and not the wall temperature as in the previously mentioned studies. Buffone and Sefiane [60] used IR imaging in capillaries with a diameter ranging from 600 to 1630 µm. They observed the signal from the open end of the capillary and were able to map the temperature of the meniscus surface in the case of evaporation of volatile liquids: ethanol, methanol (Figure 6), acetone or pentane. The thermal sensitivity and spatial resolution of their setup were 20 mK at 30 °C and 30 µm, respectively. With the strong simplifying assumption of the same emissivity for all liquids, they were nevertheless able to compare the temperature distribution along the meniscus of different volatile liquids for various temperature gradients.

### 2.3. Applicability of IRT to Gas Microflows and Current Limitations

The IR imaging technique has been widely employed in gases for leak detection [61,62] and to measure temperature. For example, Safitri and Mannan [63] have detected the leak and studied temperature by directly measuring the IR signal of methane gas, which is the major constituent in liquefied natural gas (LNG). The aim of this study was to determine the concentration and temperature of LNG vapor plumes generated by spills of LNG on concrete and water (in an open environment). Initial experiments were carried out to study the emissivity of methane in the range of 110–300 K. Subsequently, the temperature of methane gas was measured in this range. The uncertainty of the measurements has not been explicitly reported, but the authors emphasized the fact that the main uncertainty is linked to the poor knowledge of gas emissivity.

The main issue for direct gas IRT is the signal dependency on the gas emissivity, which itself is a function of temperature and is not always known with great accuracy. Moreover, only certain gases can emit energy in the infrared spectrum, but this emission is restricted to a very narrow spectral band, especially at room temperature [54]. In addition, temperature differences in a gas induce a change in the local density, and consequently changes the apparent emissivity of gases, and this phenomenon is amplified inside microscale systems.

In experiments, the IR camera is usually placed at a distance from the source whose temperature is of interest. Certain gases, such as CO_2_ and H_2_O, absorb IR wavelengths. The presence of these gases in the measurement path alters the IR signal, resulting in an error in the temperature estimation. Also, the concentration of H_2_O vapor in the atmosphere is a function of local relative humidity. However, other gases, such as N_2_, O_2_ and Ar do not absorb in the IR range, therefore having these gases in the measurement path would make the temperature measurements more reliable [64,65].

Currently, there have been very few attempts to measure temperature inside gas microflows using IRT, and researchers mainly focus on the wall radiation itself. For example, Kumar et al. [66] have developed thin Indium Tin Oxide (ITO) coated sapphire sensors with the objective of non-intrusive surface temperature measurements involving gas microflows. They were able to quantify the apparent emissivity of an ITO layer in the temperature range of 5 to 75 °C.

**Figure 7:**
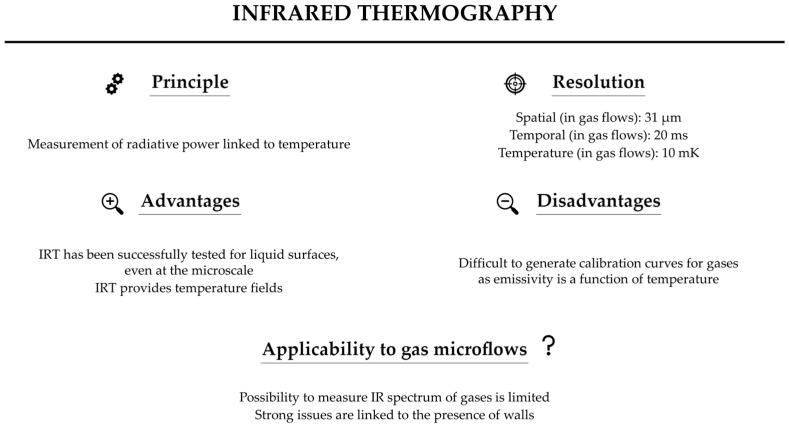
Summary of the main features of infrared thermography properties. Values of spatial and temporal resolutions are from [67], and value of temperature resolution is from [61].

To conclude this section, Figure 7 summarizes the features of the IR technique for measuring gas temperature. Researchers have reported temperature and spatial resolutions of about 10 mK [61] and 31 µm [67]. A temporal resolution of 20 ms is quite easy to achieve [67]. If, in principle, the IR technique can be employed to measure the temperature of gases, the implementation of the technique is not straightforward, since some of the key parameters, such as emissivity, are functions of temperature. In addition, for confined gas flows, the close presence of the walls that emit their own radiation considerably limits the possibility of IRT of the gas itself. These limitations have strongly restricted the usage of IR techniques to directly measure temperature in gases, particularly in internal gas microflows.

## 3. Liquid Crystal Thermography

### 3.1. Principle of Thermography with Liquid Crystals

Thermochromic liquid crystals (TLCs) are cholesteric or chiral nematic liquid crystals which exhibit an intermediate phase of matter (mesophase) between pure liquids and pure crystalline solids, whose structure, in terms of angular orientation and position of the different molecules, varies with temperature. When illuminated by a white light, depending on their temperature, TLCs reflect visible light at different wavelengths, i.e., with different colors ranging from red at the lower limit to violet at the upper limit of their working temperature. This process is reversible and reproducible. Therefore, a relation can be established between the color reflected by the crystals and their temperature [68,69].

**Figure 8:**
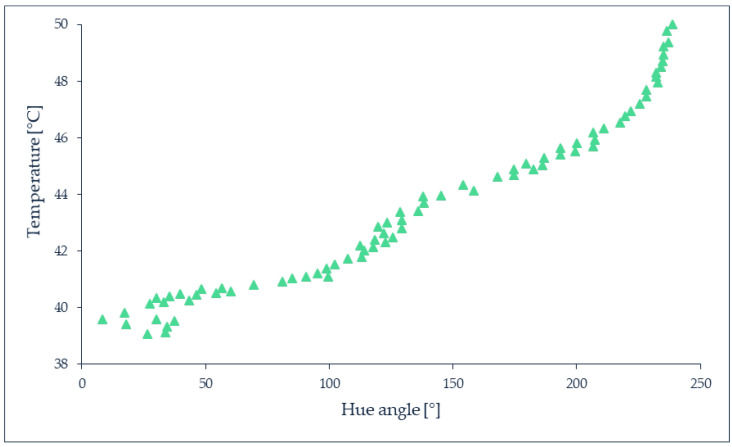
Typical calibration curve of TLC. Data from Muwanga and Hassan [73].

TLCs require calibration to relate the temperature to the hue value, which identifies the dominant wavelength of the reflected light, on a 360° scale in the HSL (hue, saturation, lightness), HSV (hue, saturation, value) or HSI (hue, saturation, intensity) color models. Figure 8 shows a typical calibration curve obtained with TLCs. Many factors such as the background surface reflectivity, color, transparency, the viewing angle between the observer and the incident light [68,70], the film thickness when TLCs are coated on a surface, the measurement and image processing techniques, must be taken into consideration during the calibration process, as summarized in [69,71,72]. The color-temperature relationship depends on the composition of the TLC, with red always being relative to the lowest temperature and blue to the highest one. Depending on the employed TLC, the red starting color can be associated to a temperature ranging between −30 and 120 °C with shifting colors bandwidths between 0.5 and 20 K [69], according to the combination choice of commercially available TLCs, such as those proposed by LCR Hallcrest Ltd. (Flintshire, UK).

**Figure 9:**
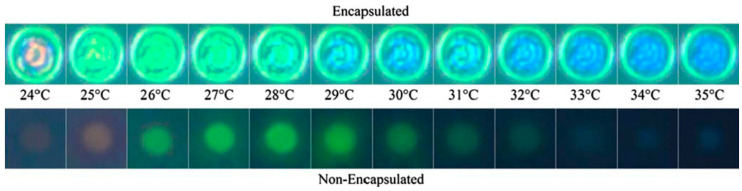
Raw color images of encapsulated TLC particles (top) and non-encapsulated TLC particles (bottom) at various temperatures, obtained by Segura et al. [76]. Article published with open access.

TLCs can be deposited as a layer on a surface or used to produce encapsulated or non-encapsulated temperature-sensitive tracer particles to seed a fluid. In the encapsulated version, an external polymer coating is present around the TLCs droplets. Therefore, they can form particles or protective microcapsules whose size can vary from the order of micrometer to millimeter and are usually in the range of 5 to 10 µm. The polymer shell protects the TLC from external contamination, such as water, but causes light aberrations and distortions in the detected color, which increases the uncertainty on the temperature measurement [74]. Some researchers have shown that non-encapsulated TLCs exhibit better color response with a higher signal to noise ratio [75]. However, because of the absence of an external protective coating, non-encapsulated TLCs can be contaminated by dust and solvent, which alter their light response and reduce their lifetime. Moreover, the exposure to UV-light deteriorates the luminescence properties of the particles over time, which may result in a color difference between the obtained signal and the initial calibration curve. Figure 9 shows the color response of encapsulated and non-encapsulated TLCs [76].

Park et al. [74] analyzed the uncertainty on the temperature measurement inferred from a single encapsulated particle. The authors point out that the main uncertainty is due to the low repeatability in manufacturing TLC particles. Even for the same temperature and illumination conditions, the reflected light varies from particle to particle. Moreover, in the case of encapsulated TLCs, the polymer shell causes a refraction of the reflected light that depends on the thickness of the coating. The authors estimated an uncertainty of 5–20%, depending on the range of working temperature, when the temperature measurement is based on a single particle. For this reason, they proposed a procedure of particle averaging for increasing the accuracy of the measurement, while reducing its spatial resolution. The same trade-off between temperature uncertainty and spatial resolution is mentioned by Basson and Pottebaum [77], who reported an in-plane spatial resolution of the order of 1 µm. This resolution was limited by the minimal size of TLC particles for which the color play is retained.

The response time, i.e., the time required by the crystal to attain a new configuration due to a slight change in temperature, is also an important parameter which is dependent on the chemical composition of TLCs [72]: it is of the order of a few ms for chiral nematic TLCs [69,78], and around 0.015 s for a cholesteric TLCs [79].

### 3.2. Applications to Liquid Microflows

Several researchers have adopted TLCs for the thermal design of micro polymerase chain reaction (PCR) systems [75,80,81]. For instance, Chaudhari et al. [75] used encapsulated TLCs to measure temperature in microfabricated arrays of 2 µL PCR vessels. Temporal and spatial temperature variations in the range of 55 to 95 °C with a resolution of 0.1 K were measured in their study. Similarly, Noh et al. [81] analyzed the temperature distribution inside the 1 µL microchamber of a micro-PCR chip during in situ temperature control using two encapsulated TLCs. The TLCs’ average capsule size was of the order of 15 µm and a temperature resolution of less than 0.3 K was obtained by averaging the hues of 64 pixels per point, corresponding to a size of each measurement point of 200 × 200 µm^2^. Hoang et al. [80] demonstrated dynamic temperature measurement, i.e., reported temperature versus time in a microfluidic chamber for PCR of 90 µm in depth and 1.5 mm in radius, using microencapsulated TLC. This was achieved by analyzing reflected spectra of TLCs as a function of time and permitted the fine tuning of the temperature controller to reduce overshoots / undershoots during temperature transitions.

**Figure 10:**
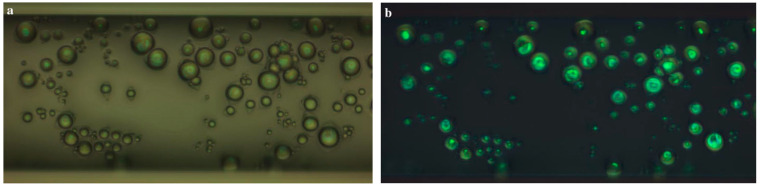
TLC particles in a microchannel with water as the working fluid, (**a**) at standard illumination, and (**b**) with circular polarization filtering, from the work of Basson and Pottebaum [77]. Reprinted by permission from Ref. [77], Experiment in Fluids, Copyright © 2022 Springer-Verlag.

Basson and Pottebaum [77] studied the temperature of water at rest using encapsulated TLCs by imposing a linear temperature gradient along a microchannel with a 100 × 100 µm^2^ cross-section. The slurry of particles was properly filtered in order to retain only TLC droplets with a diameter between 1 and 10 µm. To improve the signal to noise ratio in their study, they adopted circular polarization filtering (CPF), as shown in Figure 10a,b. A left-handed circularly polarized (LCP) light is reflected by the wall surfaces as a right-handed circularly polarized (RCP) light. The light reflected by TLC particles remains LCP, and thus CPF is used to discern between RCP reflections from the wall surface and LCP reflections from the TLC. Their working temperature range was 36.3 to 43.7 °C, and they reported an uncertainty in measured temperature between 0.4 and 2.4 K.

**Figure 11:**
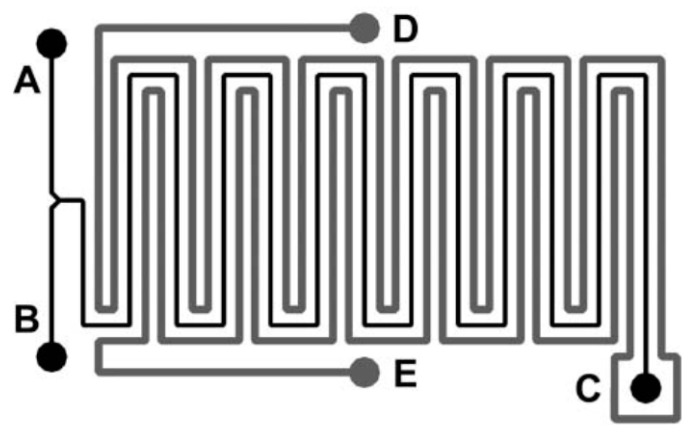
Arrangement of two microchannels from Iles et al. [82]: the reaction channel A/B-C and a collateral microchannel D-E filled with encapsulated TLCs for temperature mapping of the Reimer-Tiemann reaction in a meandering microchannel. Reprinted by permission from Ref. [82], Lab on a Chip, Copyright © 2022 The Royal Society of Chemistry.

Iles et al. [82] used encapsulated TLCs for thermal optimization of the highly exothermic Reimer-Tiemann reaction in a microreactor made of a meandering microchannel (Figure 11). The encapsulated TLCs were not seeding the fluid of the Reimer-Tiemann reaction, but were placed in a second microchannel D-E, collateral to the meandering microchannel A/B-C where the reaction took place. The TLC beads were in an aqueous slurry that offered the same thermal properties as the fluid in the reaction channel. The temperature range in this study was from 60 to 65 °C, with a resolution of about 0.4 K. To allow for precise temperature monitoring, the reflectance spectra of the TLCs were acquired using a high sensitivity fiber optic spectrometer (AVS-S2000, Avantes B.V., Apeldoorn, The Netherlands) placed perpendicular to the TLCs.

In order to reduce the uncertainty of temperature measurement in individual encapsulated TLC particles, Segura et al. [76] employed non-encapsulated TLCs produced by Shirasu porous glass (SPG) membrane emulsification, which resulted in a narrow size particle distribution approximately 13 µm in diameter. A multi-variable calibration procedure based on all three HSI color components was used to achieve very low uncertainty levels (0.65 K) in the temperature estimation of individual particles in the range of 24 to 37 °C, opening the door to simultaneous temperature and velocity tracking measurements.

In order to estimate the reliability of non-encapsulated TLCs in flows with high shear rates, experiments in controlled temperature and flow conditions were carried out by Puccetti et al. [83]. Three different types of TLC materials with a temperature range from 20 to 30 °C were studied. Experiments were carried out in a microchannel 500 μm in width, 200 μm in height, and 25 mm in length. The study did not reveal any observable color change up to a shear stress value of approximately 0.4 Pa. However, for shear stress values higher than 0.08 Pa and up to 4 Pa, the number of destroyed particles increased proportionally with the shear stress. Nevertheless, the authors showed that the color response of non-destroyed particles remained reliable.

### 3.3. Applicability to Gas Microflows and Current Limitations

TLCs, coated as thin films on a surface, have been widely employed to study the wall surface temperature, both in liquid and gas flows. For example, Muwanga and Hassan [73] coated the walls of a microchannel with non-encapsulated TLCs, 254 µm in diameter, in which distilled water was flowing, to measure local heat transfer coefficients and obtained wall surface temperature with a spatial resolution of 183 µm and an uncertainty of 0.1 K. At macroscale, several examples of the use of non-encapsulated TLC films to measure local temperature on walls and heat transfer coefficient in gas flows can be found in the review of Ireland and Jones [84] and in more recent studies (e.g., [85] or [86]). Considering the progress made on the spatial resolution of the technique (below 1 µm as reported by Muwanga and Hassan [73]), it could be applied at microscale for gas flows. It should be noted, however, that coating is not trivial. Researchers have reported practical problems, such as the difficulty in obtaining a continuous coating of constant and controlled thickness on the surface of interest, as well as a contaminant free area, erosion of coating with time, and formation of air pockets [73]. In addition, the deposition of TLCs on walls is supposed to provide information on the wall temperature or on heat transfer at the wall. However, due to the presence of TLCs, the initial surface conditions and physical properties of the wall will change. In addition, the temperature information provided by the TLC with this approach is a combination of wall and near wall gas temperature.

Nevertheless, by coating a 10 µm thin layer of non-encapsulated TLCs on the backside of a very thin (10 µm) stainless steel foil, Sodtke et al. [87] were able to obtain, under 1-g and low-g conditions, and with an uncertainty of less than 0.5 K and a high spatial resolution of the order of 1 µm, the wall temperature distribution close to the micro-region of a vapor bubble in nucleate boiling, i.e., on the tiny thin liquid film area where the liquid–vapor phase interface approaches the wall material.

**Figure 12:**
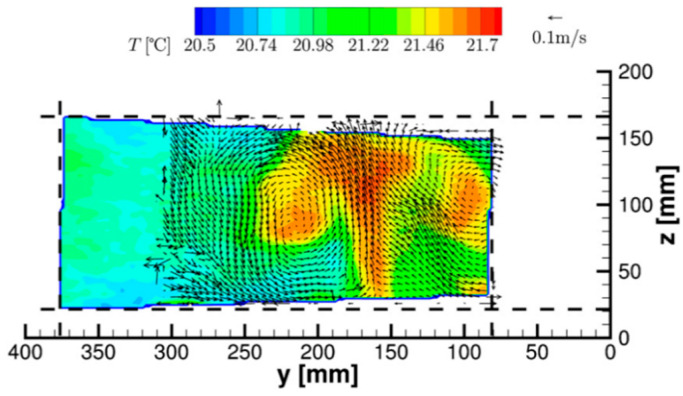
Instantaneous temperature contours and velocity vectors in a vertical plane over the heated wall of a cuboidal convection sample, from Schmeling et al. [89]. For the sake of clarity, only every second vector is shown in each direction. Recorded for a Prandtl number Pr=0.71, a Reynolds number Re=0 and a Rayleigh number $Ra=9\times 10^{7}$. Reprinted by permission from Ref. [89], Measurement Science and Technology, Copyright © 2022 IOP Publishing Ltd.

Only a few studies mention the possible use of TLCs seeded in gas flows for temperature mapping. In the works of Schmeling et al. [88,89], unencapsulated TLCs with a diameter of about 7–10 µm were used as tracer particles for the simultaneous measurement of temperature and velocity by particle image velocimetry (PIV) in convective air flows. Measurements were performed in a 500 × 500 × 2500 mm^3^ cuboidal convection sample in which the bottom plate was uniformly heated. In-situ calibration permitted the obtaining of 2D instantaneous temperature and velocity fields (see an example in Figure 12), with an accuracy for absolute temperatures of about 0.2 K and a time resolution of 0.25 s. The spatial resolution of the temperature map was limited to 40 µm in the measurement plane due to the need of averaging the hue values of particles in 12 × 12 pixel interrogation windows in order to improve the temperature measurement accuracy. In the out-of-plane direction, the resolution was of the order of a few mm due to the thickness of the white light sheet generated for particle illumination. Improvement of the technique, mainly in terms of precision and dynamic range, is linked, according to the authors, to a better characterization of TLC particles in air, their color play being known to deviate for dispersed particles from the bulk properties and the exact evaluation of the reaction time of the particles to temperature changes.

Despite these possible improvements and the interesting possibility of using TLC particles for simultaneous velocity and temperature measurements as already demonstrated for liquid flows (see for example the review from Dabiri [70]), the application to gas microflows remains questionable, mainly due to the limits in terms of spatial resolution linked to the size of the particles, which needs to be large enough to maintain their temperature-dependent reflection of different wavelengths, i.e., to keep an exploitable color play.

**Figure 13:**
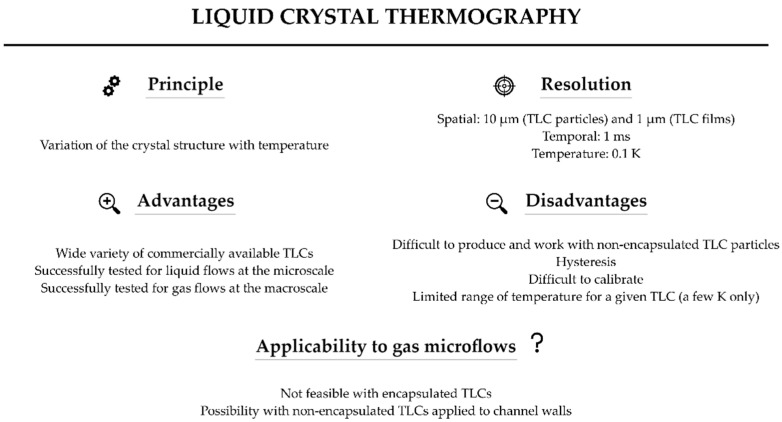
Summary of the main features of thermochromic liquid crystals. Values of spatial resolutions are from [73], value of temperature resolution is from [69,78] and value of temperature resolution is from [73,75].

Figure 13 summarizes the main characteristics of the liquid crystal thermography technique.

## 4. Temperature Sensitive Paints and Thermographic Phosphors

### 4.1. Principle of Thermography with Sensitive Paints and Thermographic Phosphors

#### 4.1.1. Surface Thermometry

**Figure 14:**
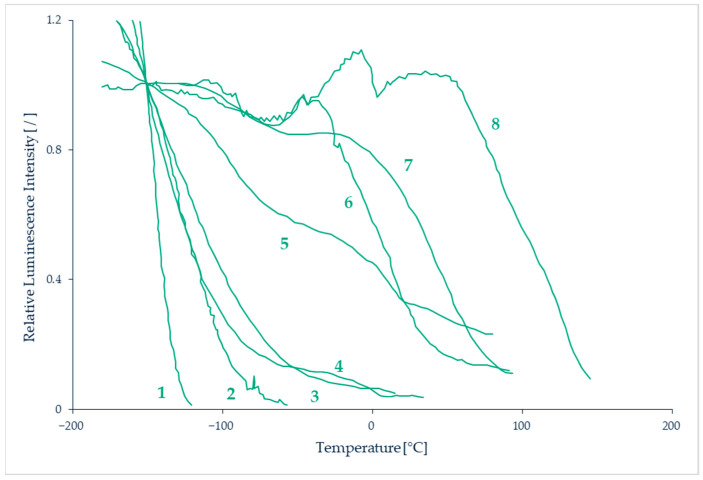
Sensitivity of several TSPs to temperature. Data from Liu et al. [92]. Luminescence intensity is plotted as a function of temperature, normalised by the intensity at a reference temperature $T_{ref}=-150\;{}^{\circ}C$. (1) Ru(trpy) in ethanol/methanol, (2) Ru(trpy)(phtryp) in GP-197, (3) Ru(trpy) in DuPont ChromaClear, (4) Ru(VH127) in GP-197, (5) Ru(trpy)/zeolite in GP-197, (6) EuTTA in dope, (7) Ru(bpy) in DuPont ChromaClear, (8) Perylenedicarboximide in sucrose octaacetate.

Temperature-sensitive paints (TSPs) are tools for mapping the temperature on solid surfaces. TSPs consist of temperature-sensitive luminescent molecules bound in a polymer after dissolving a luminescent dye in a solvent [90]. The photoluminescent molecules can re-emit light in the visible spectrum after absorbing light at a lower wavelength, often in the ultra-violet (UV) spectrum. The emitted light intensity depends on temperature, the colder molecules generally being the most luminescent ones [91]. This can be explained by the fact that, after excitation, the molecules are in an excited state. To return to their initial ground state, radiative processes directly linked to the emission of photons or non-radiative processes are involved, as illustrated by a Jablonski energy-level diagram (see further details in Section 7). Increasing the temperature promotes the non-radiative processes, resulting in a decrease of the emitted light. This phenomenon is sometimes called thermal quenching. The technique consists in analyzing either the luminescence intensity and/or its lifetime. The relationship between temperature and luminescence intensity is different for each species of photoluminescent molecule, as illustrated by the properties of some of those molecules shown in Figure 14.

The photoluminescent signal can be acquired through photo-detectors. After an appropriate signal-temperature calibration, temperature fields can be extracted. Measurable temperatures are in the range 100–430 K with an accuracy in the order of 0.5 K [93] and a spatial resolution around 2 µm [94]. The main sources of measurement uncertainty are introduced by temperature hysteresis linked to the physical and chemical properties of the paint, photodegradation of the fluorescent paint, displacement or deformation of the observed surface, or non-uniform illumination [95]. A higher signal is obtained with a thicker paint, and usual thicknesses are of the order of 100 µm.

**Figure 15:**
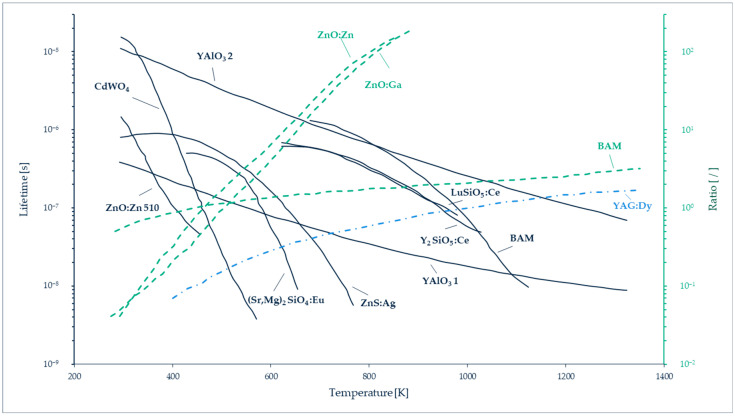
Temperature sensitivity of different types of thermographic phosphors using both two-color and lifetime analysis techniques. Data from Särner et al. [98].

An alternative technique consists in replacing the luminescent molecules with luminescent particles, called thermographic phosphor particles (TPPs). Inorganic phosphors are solid crystalline materials available in fine powder forms, made of particles with dimensions ranging from a few nm to a few µm [96]. These TTPs can be bound in a polymer paint, as in the case of luminescent particles, but they can also be directly deposited on the wall, as a thin layer on a matrix often made of ceramic or metallic material. The typical layer thickness varies between 100 and 200 µm. The main difference between TSPs and TPPs for wall thermometry is that thermography with TPPs generally exploits a phosphorescent signal rather than a fluorescent one (see Section 7 for a further explanation on the difference between fluorescence and phosphorescence). The emission spectra of the light emitted by the TPPs are temperature dependent in a wider range, 300–1800 K [97], than most of TSPs. TPPs’ signal intensity and characteristic lifetime vary as a function of the thermographic phosphor used (Figure 15), allowing a wide choice of pertinent combinations for specific temperature measurements. The luminescent signals can be processed following two approaches: by probing the intensity ratio between two emission lines in the phosphorescence spectrum (two-color or dual-wavelength method), or by analyzing the lifetime of phosphorescence (lifetime method). Typical characteristic time spans of luminescence are in the order of a few ms for various thermographic phosphors [98].

A large number of TPPs applications are related to the study of combustion phenomena. For these applications, the significant variations of temperature, i.e., several hundred K, induce a very repeatable (with a dispersion lower than 1%) and sensitive response in the luminescent intensity and life span of thermographic phosphors [99,100]. The measurement accuracy is in the order of a few K [97]. Nevertheless, some studies show a good sensitivity, of the order of 0.5 K, even at lower temperatures between 20 and 60 °C [101,102], which opens the door to other applications.

#### 4.1.2. Thermometry within the Fluid

The other major interest of TTPs is that they can be used as seeding particles inside the flow and are able to provide temperature measurements within the fluid [96]. In addition, it is then possible to combine thermometry with particle tracking velocimetry and to obtain velocity and temperature fields simultaneously. Up to now, this emerging technique has been applied to liquid, gas and two-phase flows, for example at the liquid-gas interface of vertical falling films, in heated air jets, or in internal combustion engines. A detailed analysis of these various experiments that cover a temperature from 200 to 900 K has been published by Abram et al. [96]. All the reported studies concern external flows or internal flows in devices with significant dimensions, far from those of fluidic microsystems.

### 4.2. Applications of TSPs and TPPs to Microscale

Although TSPs are currently widely used in aerodynamics, some of the first papers reporting the use of TSPs were published in the 1980s by Kolodner and Tyson [103,104], and referred to surface temperature measurements in integrated circuits. The fluorophore incorporated into the polymer film was europium thenoyltrifluoroacetonate (EuTTA), and it offered a high temperature resolution of 0.01 K and a spatial resolution of 15 µm [103], further improved to 0.7 µm [104].

**Figure 16:**
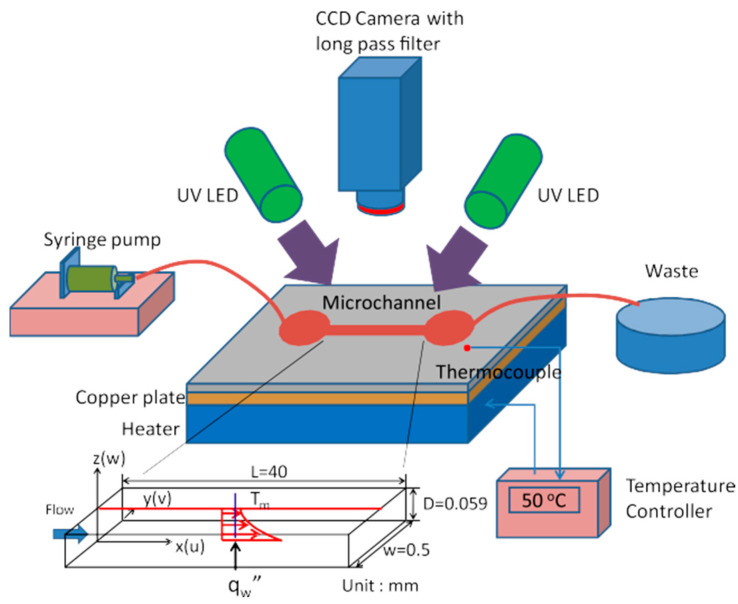
Experimental setup of Huang et al. for temperature measurement inside a microchannel by TSP thermometry [105]. Reprinted by permission from Ref. [105], Journal of Micromechanics and Microengineering, Copyright © 2022 IOP Publishing Ltd.

At the microscale, Huang et al. have used TSP thermometry to measure the temperature wall in a flow of water in polydimethylsiloxane (PDMS) microchannels 500 µm wide, 57 µm deep and 4 cm long, with single microchannel [105] or multiple parallel microchannel [106] configurations. In the single microchannel case (Figure 16), the microchannel was bonded on a heated copper plate, the temperature of which was controlled at 50 °C. The inner bottom wall of the microchannel was coated with EuTTA bound in a Polysterene (PS, Aldrich) layer. The surface temperature measurements obtained by TSP luminescence were measured, and the same EuTTA molecules were dispersed inside the water flowing in the microchannel, to provide the bulk fluid temperature along the microchannel. This bulk fluid temperature was compared to data obtained in the same conditions using Rhodamine B in deionized water as a luminescent and temperature-dependent tracer dye injected in the water flow. Some differences were noticed and attributed to microscale surface effects. This study demonstrated the capability of TSPs to measure wall temperature in a microchannel, and the possibility to use the same temperature-sensitive molecules to measure bulk liquid temperatures with small temperature variations, of the order of 0.5 K, along a small length of the microchannel, the first 4 mm, corresponding to the thermal entrance region. However, the bulk fluid temperature measurement is not done by TSP thermometry, and there is no fundamental difference when using EuTTA or Rhodamine B as a fluorophore: this technique is called laser induced fluorescence (LIF), and it is a kind of molecular tagging thermometry technique presented in much more detail in Section 7.

**Figure 17:**
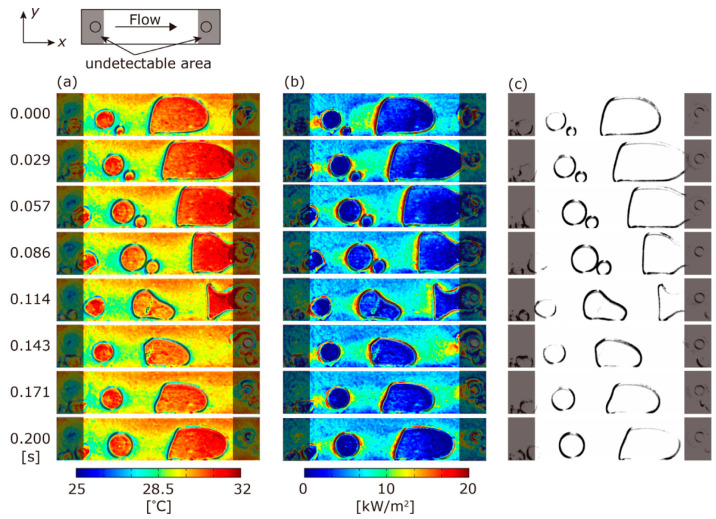
Images obtained by Matsuda et al. [107] with TSPs in FC-72 fluid boiling under upper temperature regulation (with a water channel at 40.4 °C). (**a**) Temperature distribution, (**b**) heat flux distribution, and (**c**) gas-liquid interfaces. Article published with open access.

Recently, Matsuda et al. [107] have successfully applied TSPs for detecting temperature distribution in boiling experiments inside a 50 µm deep microchannel. They demonstrated the interest of using TSPs to investigate two-phase flows as the technique allows both temperature measurements at the wall by the TSP and interface detection by visualization through the transparent TSP film. FC-72 fluid (3M, Saint Paul, MN, USA), with a saturation temperature of 55.7 °C at atmospheric pressure, was flowing in the microchannel, which was thermally regulated by means of a second channel with a temperature-regulated controlled water flow, placed on the top face of the microchannel. For this study, the temperature-sensitive molecule was 3 (Tris(1, 10-phenanthroline) ruthenium (II) hydrate (Ru(phen), Sigma-Aldrich, Burlington, MA, USA), and the polymer binder was Clearcoat UVR (AkzoNobel, the Netherlands), leading to a 2 µm TSP layer deposited on the inner top wall of the microchannel, i.e., between the microchannel and the upper water channel. Initial isothermal two-phase flows were analyzed. Although the temperature should be the same in both phases, a temperature difference of about 3 K was detected between gas and liquid phases which the authors attributed to the difference in the refractive index of the two phases. This deviation allowed the detection of the gas-liquid interfaces, and a correction was applied to obtain the real temperature in the gas. Next, boiling experiments were conducted, and the analysis provided both temperature fields, and consequently heat fluxes, and interface detection (Figure 17).

Although TPPs can have low dimensions, down to some nm, their implementation in microflows has not been published yet, and current spatial resolution is limited to 200 µm [96]. Further research in this field is necessary to apply TTP thermometry to microfluidic devices.

### 4.3. Applicability of TSPs and TPPs to Gas Microflows and Current Limitations

**Figure 18:**
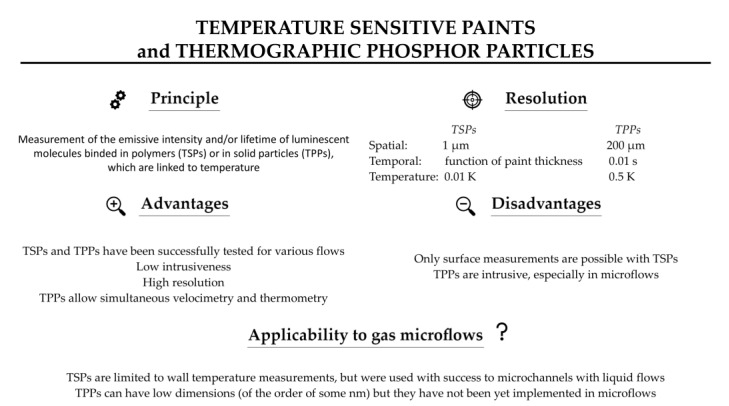
Summary of TSPs’ and TPPs’ features. Values of spatial resolutions are from [104] and [96], for TSPs and TPPs respectively; value of temporal resolution is from [96]; values of temperature resolution are from [103] and [101], for TSPs and TPPs respectively.

As far as we know, there is no study involving TSPs in gas microflows analysis. The literature survey shows that it could be successfully applied at microscale and/or for rarefied gas flows as it was the case for pressure measurements with pressure-sensitive paints (PSPs) in high Knudsen number flows [108]. However, temperature-sensitive paints can only provide surface temperatures, and the thickness as well as the roughness of the paint could be a limitation for microfluidic devices with very small dimensions, lower than some tens of µm. For pressure sensitive paints, the Langmuir Blodgett method has been implemented by Matsuda et al. [109,110] to reduce the paint thickness to a single molecular film. The obtained pressure sensitive molecular films (PSMFs) have been successfully used to observe pressure distributions at the wall of micro-nozzles in the region of the throats, 103 and 48 µm in width. The authors concluded that PSMFs spatial resolution was adapted to microscale pressure measurements in fluidic microdevices with characteristic lengths over 50 µm. Unfortunately, up to now, such a molecular film deposition technique has not been developed for temperature-sensitive luminophores. The spatial and temperature resolutions of TSPs can be high (Figure 18), and the temporal resolution, which depends on the heat fluxes and inertia linked to the thickness of the TSP, is compatible up to a certain extent with non-stationary flows analysis [107]. In gas microflows, however, heat fluxes can be limited and the response time quite low.

As previously explained, additional research is required to apply TTP thermometry to microfluidic flows, and more specifically to gas microflows.

## 5. Interferometry-Based Thermometry (IBT)

### 5.1. Principle

When two light waves are superimposed, their individual spatial and temporal sinusoidal variations interfere and these two waves can locally reinforce or cancel each other, resulting in a distribution of intensity variations; this is the so-called interference phenomenon. The wave features depend on the refractive index *n* of the medium in which the wave is propagating with a speed

(2) $$v=\frac{c}{n}$$

where *c* is the speed of light in vacuum, approximately equal to 3 × 10^8^ m s^−1^. As the refractive index of a fluid can vary with its properties (pressure, temperature, concentration of different components), the interference pattern can be exploited for thermometry purposes if the two waves go through fluid zones at different temperatures before interfering.

The interferometry of refractive index fields has thus been widely used in applications of thermal and fluids engineering [111]. In division of amplitude interferometry, a change in optical paths between two or more coherent (i.e., single-wavelength) light beams is measured, but in most of the interferometers, light issued from one single laser is divided into only two beams. One beam passes through the experimental test cell and the other beam follows a reference path with uniform known temperature and physical properties. The experimental test section experiences certain temperature, density, concentration or pressure distributions. The variation of these physical quantities results in the modification of the refractive index in the test section, leading to a variation δ in the phase of the optical beam:

(3) $$\delta =\frac{2\pi L}{\lambda_{0}}\left(n-n_{0}\right).$$

In Equation (3), $\left(n-n_{0}\right)$ is the refractive index change between the reference medium and the test cell, the thickness of which is *L*, and $\lambda_{0}$ is the wavelength of the laser beam. For the specific case of gases, the refractive index *n* in the test cell is linked to the density ρ according to the Gladstone-Dale equation

(4) n−1=kρ

where the Gladstone-Dale constant *k* is a function of the wavelength. Finally, *n* can be linked to the pressure and temperature, following the Boyle-Mariotte law for a perfect gas:

(5) p=ρRT,

where *R* is the specific gas constant. When pressure *p* is known and uniform in the test section, δ then provides an indirect measurement of temperature *T*, according to Equations (3)–(5).

In practice, after crossing the reference and test sections, the two light beams are recombined, resulting in the emergence of interference patterns or white and dark fringes (interferograms). In case the interference pattern which has emerged is due to a two-dimensional temperature field at uniform pressure, each fringe represents an iso-density, and, consequently, an isothermal region. There are a variety of methods by which the temperature at each fringe can be evaluated, thereby providing a mapping of the temperature field. Interferometry requires two principal sets of instruments [50,112,113]. The first one regroups the various optical instruments required to generate an interference pattern. The second one is dedicated to the observation and processing of interferograms.

**Figure 19:**
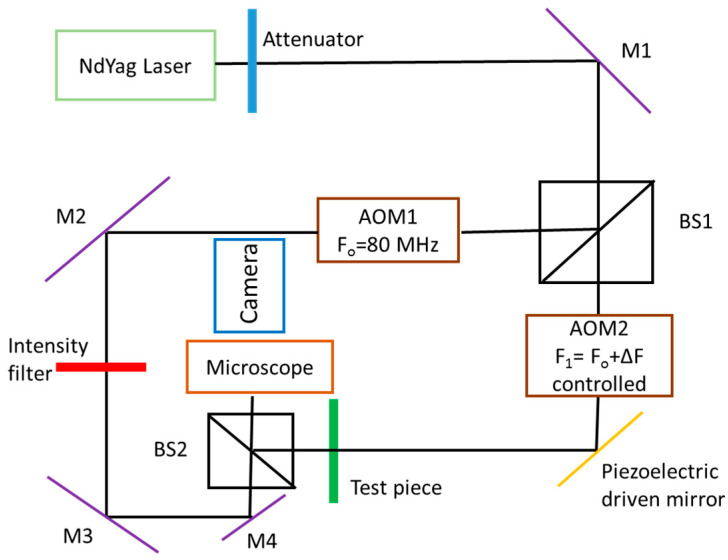
Typical interferometry setup for microfluidic applications, adapted from Garvey et al. [50].

Figure 19 shows a typical Mach-Zehnder interferometric setup implemented for microfluidic measurements. A neodymium-doped yttrium aluminum garnet (Nd:YAG) laser source provides the laser beam. Mirrors M1, M2, M3 and M4 reflect the laser beam in appropriate directions and the beam splitter BS1 separates the initial beam into a reflected component and a transmitted component. The beam splitter BS2 recombines these two beams. A piezoelectric driven mirror can introduce phase stepping in the transmitted beam. Acousto-optic modulators, AOM1 and AOM2, can introduce signal heterodyning, which is a process to create interferences between two signals from the same source shifted by a frequency difference Δ*f*. The control of the signal phase or frequency is used for applying the phase measurement interferometry (PMI) technique, which allows us to measure the phase shift produced by the change in the optical path distance from multiple intensity patterns. The beam recombined by BS2 passes through a microscope lens to magnify the interference pattern, and it is captured with a camera. The interferometric setup shown in Figure 19 was developed by Garvey et al. [50], and has been used to compare phase stepping and heterodyne retrieval techniques for extracting the phase change between the reference beam and the beam crossing the test section. It was demonstrated that the heterodyne phase retrieval technique allows a six times higher resolution than the classic phase-stepping retrieval technique.

### 5.2. Applications to Liquid Microflows

In the work of Garvey et al. [50], the setup shown in Figure 19 was implemented to analyze the mixing of water with a 0.2 mol/L NaCl solution in a T-junction of 500 × 500 µm^2^ square microchannels, and provided concentration profiles along the microchannel with a spatial resolution of 8.9 µm. The authors found a rather good agreement with theoretical predictions. Although the study was dedicated to concentration field analysis, a short discussion about the possible use of this setup for measuring temperature fields in a liquid suggested that a resolution of 0.1 K could be reasonably achieved with the heterodyne technique.

**Figure 20:**
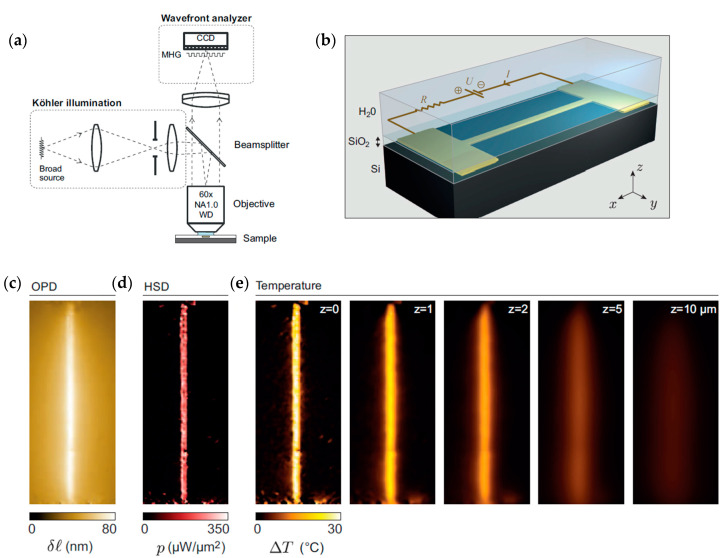
(**a**) Schematic of the experimental setup developed by Bon et al. [114]; (**b**) gold microwire embedded in a layer of SiO_2_ and connected to two gold electrodes; (**c**) optical path difference (OPD) image; (**d**) heat source density (HSD) image obtained from the OPD; (**e**) temperature distribution at various heights from *z* = 0 (microwire position) obtained from the HSD images. Adapted and reprinted by permission from Ref. [114], Applied Physics Letters, Copyright © 2022 AIP Publishing LLC.

Bon et al. [114] developed an interferometry technique, abbreviated as TIQSI, for temperature imaging using quadriwave lateral shearing interferometry. With this technique, they measured the three-dimensional temperature distribution around a heated gold microwire of 40 nm thickness, 1 µm width and 80 µm length, immerged in a thin water layer. The experimental arrangement is shown in Figure 20a,b. An optical path difference (OPD) due to thermal induced variation of the surroundings refractive index of water was measured with a wavefront analyzer, and the image is shown in Figure 20c. A numerical approach based on Green’s function was developed to evaluate the 3D temperature distribution. The ambient temperature in this study was 23 °C, and the heated wire had a temperature of about 60 °C. The corresponding heat source density (HSD) images, and the temperature distribution at various heights up to 10 µm from the microwire are shown in Figure 20d,e, respectively. A comparison with a numerical simulation showed a rather good agreement. The spatial resolution, however, was limited to 450 nm by the diffraction limit, and the steepest expected temperature gradients were not experimentally captured.

### 5.3. Applications to Gas Macroflows

Interferometry has been applied to study various phenomena involving gases, such as combustion heat transfer, sometimes in transient conditions, but most of these studies were at macroscale [111]. Otherwise, researchers have expressed concerns over the high cost involved in the successful implementation of the interferometry technique. This is usually due to the requirement of high-quality optics. To tackle this aspect, Forno and Whelan have developed a technique known as digital moiré subtraction (DMS) [115]. In this process, which consists in combining intensity distributions of two dissimilar grid patterns, the optical aberrations are corrected. DMS has been further implemented by Newport et al. [116] for the analysis of electronics cooling in enclosures. The implemented Mach-Zehnder interferometric arrangement was able to provide a field of view of 140 mm.

**Figure 21:**
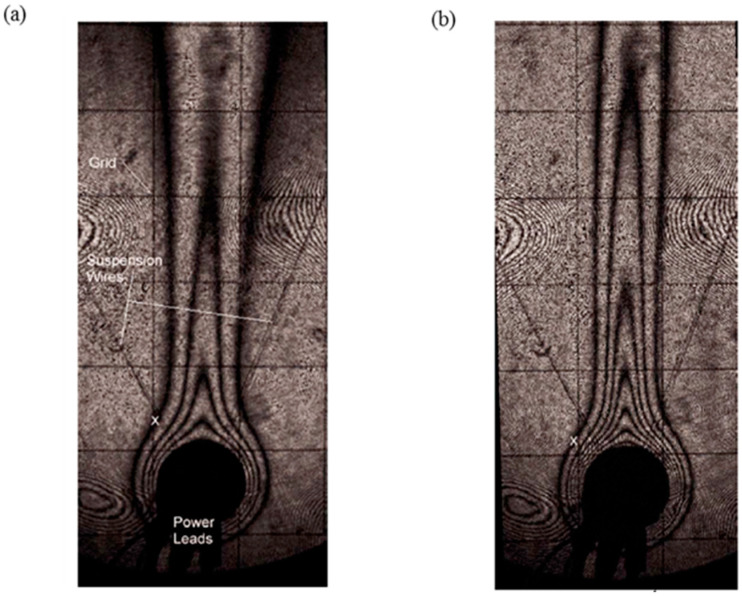
DMS interferograms obtained by Newport et al. [116] in a free convective air flow around a heated cylinder, for (**a**) $Ra=6.8\times 10^{3}$, and (**b**) for $Ra=1.4\times 10^{4}$. Reprinted by permission from Ref. [116], Journal of Electronic Packaging, Copyright © 2022 ASME.

The DMS technique was first successfully compared with classical interferometry by measuring free convection temperature fields in air around a heated cylinder 20 mm in diameter. Figure 21a,b show the DMS interferograms obtained around the isothermal heated cylinder for different values of the Rayleigh numbers *Ra*, which represent the ratio of the time scale for diffusive thermal transport to the time scale for free convective thermal transport. The uncertainty of the extracted data was, at worst, 0.7 K.

The technique was then applied to investigate the thermal interaction between 2D components representing ball grid arrays (BGAs) mounted on a vertical printed circuit board (PCB). In these different macroscale experiments, the gas was neither in a confined environment, nor in rarefied conditions.

### 5.4. Applicability to Gas Microflows and Limitations

Newport et al. [113] carried out a detailed study to assess the factors limiting the wide applicability of interferometry to fluids at microscale. They based their study on noise and error analysis to assess the limitations of the interferometric approach to measure the temperature in different channels, varying dimensions and employing gases and liquids as working fluids.

Temporal noise, identified as one of the limiting factors at microscale, is defined as the variation in the system’s phase with time [113]. Conduction by device materials significantly affects the thermal field and the velocity field in the region external to the boundary layer. Therefore, conduction within the device must be considered in a design of the prototype or numerical models, in order to avoid unwanted phase accumulation [111]. Other factors, such as small phase shift signal, also serve as obstacles in fully exploiting interferometry [112]. Additionally, for gas flows, strong issues linked to low density (compared to liquids) should be considered. There is approximately a thousand-fold difference between the density of gases and liquids, and this difference in density increases with rarefaction, thereby complicating the task of temperature measurements. The study from Newport et al. [113] revealed the resolution limits of interferometry, which is directly linked to the length travelled by the light through the fluid portion, the temperature of which should be measured. The smallest measurable phase change was determined from a spatial noise analysis and was estimated at ±0.3 rad. Considering a channel with a depth *d*, the resolution of the average temperature measured through the depth of the channel is inversely proportional to *d*. In a minichannel with d=1 cm, a resolution of 0.01 K may be achieved in oil, of 0.1 K in water, and achieving a temperature resolution of 1 K in air at atmospheric pressure would require a lower phase uncertainty. These resolutions should be multiplied by 10 if d=1 mm, and reaching a rarefaction regime would require a depth, or a pressure, 1000 times lower, multiplying the resolution with the same factor of 1000. Also, even if in theory, temperature resolutions as low as $10^{-5}\;K$ have been reported for optical interferometry [117]; this resolution is directly linked to the scale of the device. The conclusion is that, even with a drastic improvement of the smallest measurable phase change and the use of a multiple number of passes through the channel, a resolution of 1 K is unachievable for microflows, especially in the case of gases.

**Figure 22:**
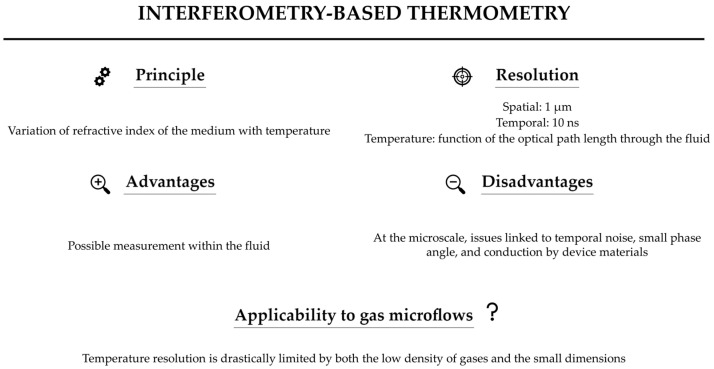
Features of interferometry-based thermometry. Values of spatial and temporal resolutions are from [117].

Figure 22 summarizes the main characteristics of interferometry.

## 6. Light Scattering Thermometry (Raman and Rayleigh)

### 6.1. Principle

Light scattering by matter can be elastic or inelastic. In a classical interpretation, when a molecule (a polarizable ensemble of positive and negative charges) is hit by an oscillating electric field, the induced oscillating dipole moment radiates in almost all directions. Most of this radiation bears the same frequency as the incident field, but it is accompanied by some weaker components at beat frequencies with those of the internal (rotational and vibrational) molecular motions. The former phenomenon is the elastic, or Rayleigh, light scattering, while the latter constitutes the inelastic, or Raman, scattering.

**Figure 23:**
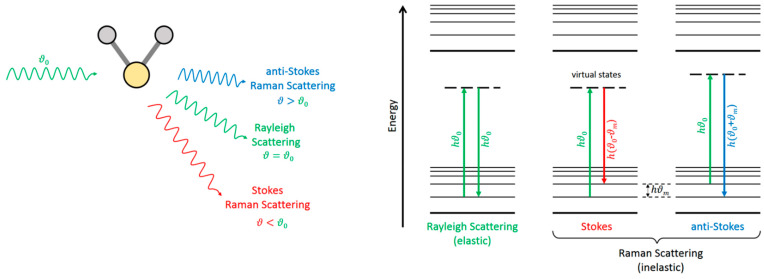
Illustration of Rayleigh and Raman (Stokes and anti-Stokes) scattering. In this figure, ϑ represents the wave frequency and h is the Planck constant. Subscript 0 refers to the incident photon.

In the quantum interpretation, an incident photon is annihilated by the interaction with the molecule, and a different photon is (re)emitted along a different direction, in a process much faster (<10^−15^ s) than fluorescence (see Section 7). In the Rayleigh scattering, both incident and scattered photons have the same frequency, and the molecule ends in the same energy level (Figure 23). But in the Raman scattering, the frequencies of the incident and the scattered photons are different, and thus the molecule undergoes a transition from an initial to a final state with different energies. Depending on whether the final energy of the molecule is higher or lower than the initial one, the Raman scattering is denoted Stokes or anti-Stokes (Figure 23).

Both Rayleigh and Raman scatterings are nonintrusive techniques that can be used to retrieve temperatures from unseeded gas flows. Rayleigh scattering is more intense than Raman, but it is almost invariably accompanied by undesired stray light, with the same frequency, from surfaces and particles. On the contrary, the rich spectral structure of Raman scattering allows for the accessing of the true molecular information in a more straightforward way, as explained below.

The Raman spectrum is the representation of the scattered intensity versus the energy shift from that of the incident photons, customarily reported in units of reciprocal wavelengths (1/*λ*) or wavenumbers. The Stokes and anti-Stokes components lie at symmetric shifts with respect to the much more intense Rayleigh peak. The integrated intensity of the Rayleigh peak is proportional to the total number density of molecules (in absence of clustering), but its spectral profile is sensitive to the gas temperature, as exploited by Filtered Rayleigh Scattering (see below). On the contrary, the intensity of any feature (vibrational band, rotational lines) in the Raman spectrum is proportional to the number density of molecules in the initial level of the transition. The local temperature can be retrieved from the Raman spectrum in at least, four ways, which are as follows, in approximate decreasing order of accuracy:

(a) Population of known energy levels,
(b) Frequency of a temperature-sensitive line,
(c) Stokes/anti-Stokes ratios,
(d) Band contours.

It should be noted that (a), (c), and (d) rely on the Raman intensity, and are outlined next.

**Figure 24:**
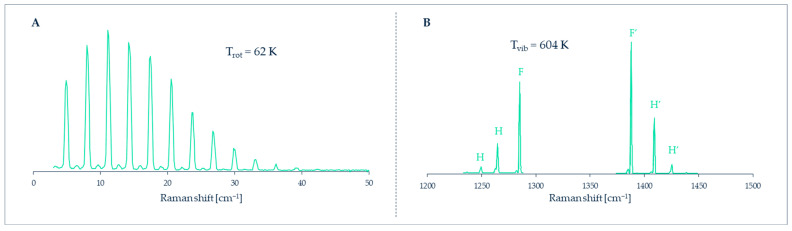
Raman spectra of supersonic jets of CO_2_ from a 0.4 mm nozzle. (**A**) Rotational Raman spectrum; the resolved rotational lines correspond to different angular momentum *J*. (**B**) Vibrational Raman spectrum; peaks labeled H, H’ are hot bands (corresponding to transitions starting from excited vibrational levels) of the F, F’ fundamental bands.

In favorable cases of gases composed of light symmetric molecules (e.g., H_2_, N_2_, O_2_, CO_2_), the individual rotational lines can be resolved in the spectrum, as in Figure 24A. The intensities of these rotational lines are related to the number density of molecules in the initial energy levels *J* of the transitions. This allows direct access to the populations $P_{J}$, i. e., the fraction of molecules in each energy level *J*, normalized as $\sum P_{J}=1$. For the Boltzmann distribution

(6) $$P_{J}\propto g_{J}\exp\left(-\frac{E_{J}}{k_{B}T}\right),$$

where $k_{B}$ is the Boltzmann’s constant and $E_{J}$ and $g_{J}$ are the energy and quantum degeneracy of the level, respectively, a macroscopic temperature *T* can eventually be retrieved from Equation (6). In some occasions, well resolved hot vibrational bands, as in Figure 24B, can be used in a similar way.

Any pair of corresponding Stokes and anti-Stokes lines (or bands), at symmetric frequency shifts from the elastic peak, connect the same two energy levels (either rotational or vibrational) of the molecule, but in opposite senses: upwards for the Stokes (S), and downwards for the anti-Stokes (aS). Thus, the ratio of their intensities *I* yields the ratio of the populations of the two levels

(7) $$\frac{I_{S}}{I_{aS}}=\exp\left(\frac{\Delta E}{k_{B}T}\right),$$

where Δ*E* is the energy gap between the two levels.

**Figure 25:**
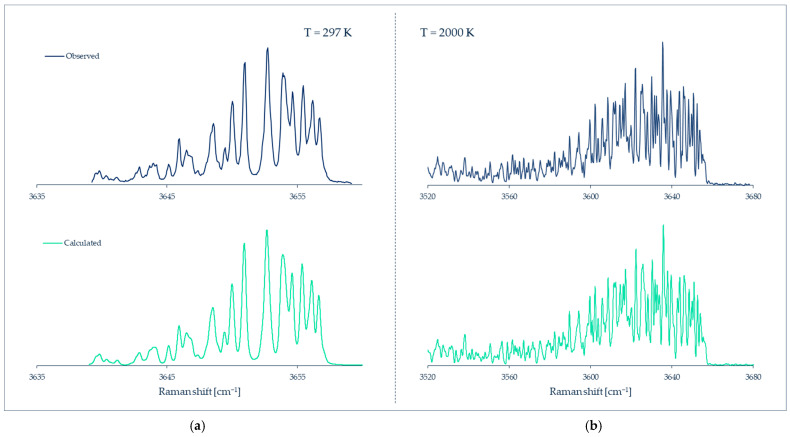
Raman spectra of the OH stretching band of gaseous H_2_O at (**a**) 297 K, and (**b**) ~2000 K, observed and simulated by Avila et al. Data from [118].

In heavier or asymmetric molecules, the rotational lines are closer in frequency and cannot be resolved; on the other hand, in liquids (and solids) rotational motions are hindered by interactions with neighbor molecules and do not give rise to discrete energy levels. The practical consequence in both cases is that discrete rotational lines cannot be observed, but only the contour/profile of a broad band, and something similar happens for most of the vibrational bands in polyatomic molecules. In all of these cases, the temperature modifies the band profiles to a greater or lesser extent, and therefore it is still possible to obtain the temperature, although less accurately, by empirical calibration or simulation of the band profile (Figure 25).

The main advantages of the Raman technique are: (i) universality, in the sense that all substances composed of molecules give rise to the Raman spectrum; (ii) high spatial resolution of a few µm; (iii) wide spectral range (>4000 cm^−1^); and (iv) long term stability and repeatability. The main disadvantage is that the Raman effect is feeble (cross sections are typically 10^−34^ m^2^ sr^−1^), and the Raman signal can be obscured by fluorescence or phosphorescence from windows or from the sample itself. Thus, a high sensitivity instrument (or long acquisition times), with good rejection of the dominant elastic scattering is often needed for the practical realization in gaseous samples, as detailed below.

The high spatial resolution of the technique is due to the extremely weak interaction between photons and molecules: the Raman signal only comes from the volume of the focused laser beam, where the irradiance is high enough for the effect to be detected. In current imaging spectrometers, this region can be reduced even further (and the spatial resolution augmented) by reading a reduced area of the bi-dimensional CCD detector.

The technique is truly non-intrusive because the Raman effect is very weak: only a tiny fraction of the molecules suffer a transition, thus the perturbation to the probed medium is negligible. However, in microfluidic devices, attention must be paid to the undesired absorption of the exciting laser beam by nearby components like windows or walls, which may lead to artificial sample heating.

For the practical realization of Raman spectroscopy, the main instrumental components are a high-power laser and a spectrometer with high sensitivity and good rejection of the elastic scattering. For the latter, double or triple monochromators are often employed, but currently they can be replaced by optical “notch” or high-pass filters in tandem with single monochromators [119]. For routine operation with gases, the laser must provide 1 to 10 W continuous wave of visible light, and the spectrometer focal length must be 30 to 100 cm, with a grating of 1200 groove/mm for good spectral resolution, and a cooled CCD detector for good sensitivity. For liquids, current compact spectrometers (~10 cm focal length) can be used as well, with 100 to 1000 mW laser power. In special favorable cases, the spectrometer can be replaced by band-pass filter(s) tuned at the wavelength(s) of interest [120]. The laser and spectrometer are complemented with the corresponding optics (and possibly optical fibers) for excitation and collection, and eventually an appropriate sample cell (microfluidic device). As with other optical-based techniques, some windows transparent to the incident and scattered radiation are needed for measuring inside a microfluidic device.

So far, we have described the original, so-called “spontaneous”, Raman scattering. There are other variants of the Raman technique, such as surface enhanced Raman spectroscopy (SERS), or coherent anti-Stokes Raman spectroscopy (CARS), with particular enhancements of sensitivity or resolution, that have been applied to liquid microflows [121,122,123]. However, the observed intensities in these variants are often compromised by reproducibility issues (SERS) or uncontrolled environmental contributions (CARS), which render retrieving the temperature [124] not as straightforward as explained above, or not possible at all.

Filtered Rayleigh scattering (FRS) [125] is closely related to Raman thermometry (RT), and shares much of the instrumentation. The true Rayleigh scattering is spectrally broadened and shifted by the molecular velocities and collisions, while the undesired accompanying light from windows and walls preserves the spectral purity of the exciting laser. If a spectrally-narrow (single-mode) laser like a seeded Nd-YAG is employed and tuned to a spectral transition of molecular iodine, much of the undesired background light can be efficiently notch-filtered by an absorption cell before the detector. The temperature of the gas can be retrieved from the filtered Rayleigh light, relaying on appropriate models and calibration references. FRS is best suited for high temperatures or high velocity flows, and has been successfully applied to the 2D temperature imaging of combustion flames [126] and supersonic flows [127], with best time resolution but less accuracy than from Raman thermometry. Although potentially attractive, to our knowledge FRS has not been applied to microflows so far, and thus it will not be considered further.

### 6.2. Applications to Liquid Microflows

There are many recent applications of Raman spectroscopy and its variants to characterizing liquid flows at the microscale. Most of the instruments, commercial or home-made, work here in a confocal configuration by using a microscope objective to both focus the exciting laser and to collect the backscattered radiation. This arrangement, which combines the high spatial resolution of microscopy with the species-dependent information of Raman spectroscopy, is also known as Raman microscopy. A review of this instrumentation, along with other considerations for Raman microscopy applied to liquid microfluidics, can be found in [128].

Most of the applications of Raman microscopy to liquid micro-flows are devoted to measuring the amount of substances (or density). Thus, composition profiles in microfluidic devices have been measured for mixing processes [129,130], diffusion through interfaces [131,132,133], or chemical reactions [134,135,136,137]. Roetmann et al. [138] and Rinke et al. [139] were able to record 2D density maps (Raman “imaging”) of mixing miniflows, with subsecond time resolution, by using pulsed (6 ns) excitation.

In turn, the true Raman thermometry of liquids in microfluidic devices has been accomplished for water or aqueous solutions. This is because the band profile of the OH stretching around 3400 cm^−1^ is quite sensitive to the temperature, at least between 3 and 72 °C [140], which can be modelled in a simple way: the logarithm of the ratio of the integrated band areas above and below some arbitrary “center” frequency is a linear function of 1/*T*, over a wide temperature range [141].

**Figure 26:**
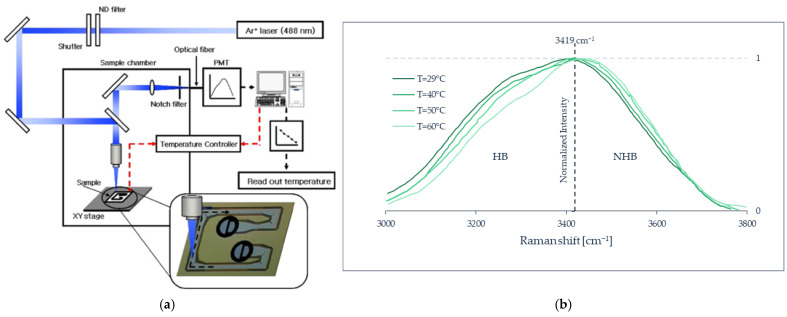
(**a**) Schematic diagram of the micro-Raman spectroscopy system used by Kim et al. Reprinted by permission from Ref. [142], Journal of Micromechanics and Microengineering, Copyright © 2022 IOP Publishing Ltd. (**b**) Changes in the OH stretching band of liquid water at different temperatures. Data from [142].

With that method, Kim et al. [142] measured the temperature distribution inside the water-filled microchannel, with a 600 × 130 µm^2^ section area, of a polymerase chain reaction (PCR) chip (Figure 26), and found that, for a 55 °C controlled chip, it ranged from 53.6 to 54.8 °C along a 13 mm path length. This demonstrates the accuracy of the measurements, as well as the actual inhomogeneity of the water temperature inside the microchannel. Ewinger et al. [143] extended the thermal range from 30 to 58 °C for a 0.4 × 0.2 mm^2^ microchannel, and demonstrated a spatial resolution of 15 µm in width and 25 µm in depth, with an accuracy of ±1.2 K. Brinatti Vazquez et al. [144] were able to measure temperature differences up to ~5 K along the depth of a 0.5 × 0.15 mm^2^ microchannel.

Kuriyama and Sato [120] were able to record a 2D map of temperatures (thermal imaging) over a 820 × 820 µm^2^ field of the mixing of hot and cold water at a T-junction. They were able to visualize the non-uniform temperature distribution with a spatial resolution of 12.8 × 12.8 µm^2^. The same authors intended to retrieve the flow velocity from heating a spot in the liquid with a laser pulse, and to track its downstream evolution by Raman thermal imaging [145], and they estimated that fluid velocities up to ~30 mm s^−1^ could be measured for thermal differences larger than 9 K.

It must be stressed that liquid water is a very fortunate case for Raman thermometry, since the Raman signal is quite sensitive to the temperature and relatively easy to calibrate, but this is not the case for many other liquids. On the other hand, water is the most common liquid for microfluidics applications.

**Figure 27:**
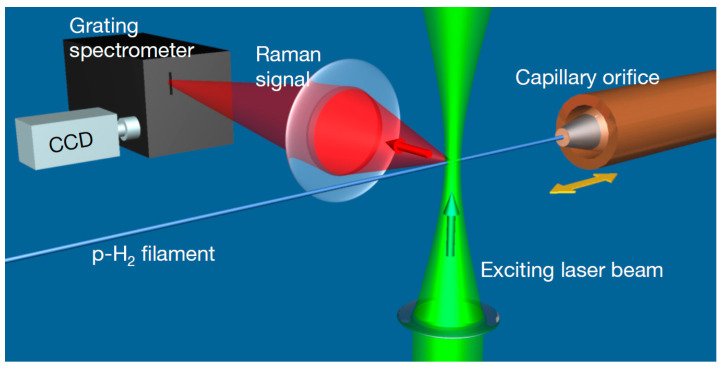
Schematic view of the experimental setup for Raman spectroscopy of liquid microjets used by Kühnel et al.

So far we have considered confined flows. However, Raman thermometry has also been applied to unconfined “free” liquid jets. Kühnel et al. [146] injected 5 µm microjets of liquid hydrogen into a vacuum from a cryogenic nozzle at 17 K (Figure 27). The liquid jet cooled rapidly by evaporation until it eventually crystallized. The temperature of the undercooled liquid jet, down to crystallization, was measured by the peak frequency of the Raman vibrational band [147].

In a somehow similar experiment, Goy et al. [148] injected a train of perfectly regular water microdrops, ~6 µm in diameter, into a vacuum, and tracked their supercooling and crystallization. In this work, the temperature of the liquid water drops was measured from the Raman spectrum from the familiar ratio of high and low frequency semi-intensities of the OH stretching band extrapolated to temperatures below 0 °C [141].

### 6.3. Applicability to Gas Microflows and Current Limitations

To the best of our knowledge, Raman thermometry has not yet been applied to gas flows in microfluidic devices. So far it has been successfully applied to free (i.e., unconfined) flows such as miniflames [149] or supersonic gas microjets of H_2_, N_2_, O_2_, CO_2_, H_2_O (gas) and some of their mixtures [150,151,152,153,154]. In N_2_ [155], O_2_ [156], and CO_2_ [157,158] the temperature was determined through the rotational populations from the rotational Raman spectra, while in H_2_ the rotational populations were measured from the rotationally-resolved vibrational Q-branch [159,160]. For H_2_O (gas), separate rotational lines in the vibrational Q-branch cannot be fully resolved, and the temperature was retrieved from the simulation of the Q-branch profile [161].

The same approaches can, in principle, be applied to gas microflows inside glass microchannels. The technique is better suited for light gases made up of molecules such as H_2_, N_2_, O_2_, CO, CO_2_, CH_4_, NH_3_, or H_2_O. The vibrational bands of such molecules lie at high frequencies, are separated from the Rayleigh peak, and their rotational lines can be resolved in the Raman spectrum, allowing for an accurate local temperature measurement. For vapors made of small organic molecules like butane, ethanol, or acetone, the temperature can, in principle, be retrieved from the Stokes/anti-Stokes ratio of low-frequency vibrational bands, or from temperature-sensitive band profiles.

With regard to potential limitations, the pure rotational Raman spectrum lies close to the Rayleigh peak, and therefore can be difficult to observe in microfluidic devices in the presence of a lot of elastic stray light, or even blocked by the rejection filter. On the other hand, the Stokes/anti-Stokes ratio method is best suited, at room temperature, for bands up to ~500 cm^−1^, otherwise the anti-Stokes component becomes too weak. Such low frequency bands lie not far from the elastic Rayleigh peak, and thus might be difficult to observe; of course, at higher temperatures, band pairs at higher frequencies can be successfully employed.

**Figure 28:**
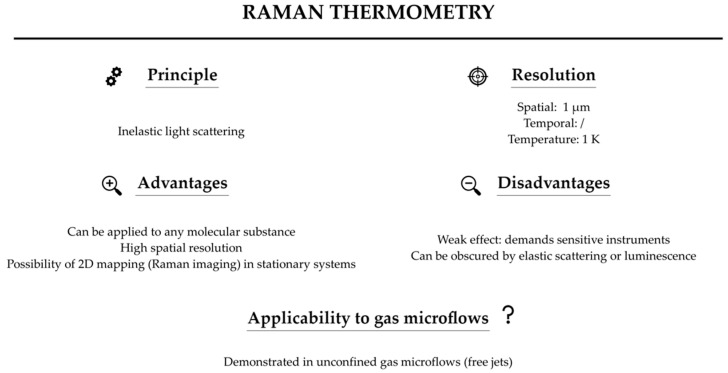
Summary of Raman thermometry features.

To recap, despite the aforementioned limitations, Raman spectroscopy is a powerful, non-invasive technique, capable of measuring local temperatures in gas microflows, with high spatial resolution (Figure 28). This great potential is expected to be realized in confined flows within microfluidic devices in the next few years or decades.

## 7. Molecular Tagging Thermometry

### 7.1. Principle

**Figure 29:**
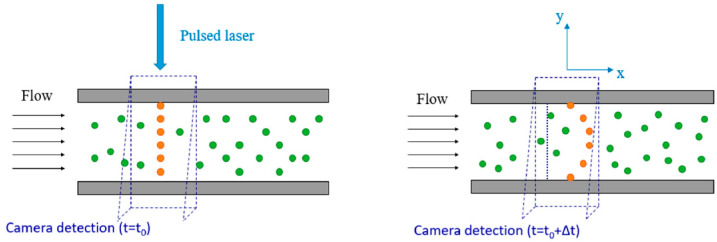
Basic principle of 1D-molecular tagging velocimetry (MTV) by direct phosphorescence for gas flowing in a plane channel from left to right, schematized by Fratantonio et al. [162]. Article published with open access.

Molecular tagging (MT) represents a technique in which the photoluminescence of tracer molecules seeded into a fluid flow is observed with the goal of measuring local fluid properties such as velocity, temperature, molecular concentration, density or pressure. So far, the technique has been mostly employed for velocimetry (MTV) and thermometry (MTT). Photoluminescence represents the ensemble of intramolecular processes that occur when a molecule that absorbs a photon re-emits part of the energy back, after some time, in the form of light in the visible spectrum. The basic principle of the MT technique consists in exciting the tracer molecules by means of a light source. After excitation, a radiative decay at the electronic level occurs in the tracer molecule, which generates the photoluminescent effect. Velocity fields can be measured by tracking the molecular displacement of the tagged molecules (Figure 29), while temperature fields can be measured by analyzing the luminescent intensity and temporal decay of the signal. Eventually, both velocimetry and thermometry results could be retrieved from the same luminescent signal.

Some typical tracer molecules used in the MT of gas flows include acetone and diacetyl. In addition to these molecules, studies have also been conducted for gas flows on toluene, tert-butyl nitrite, sodium, strontium, OH, and O_2_, to name but a few [163]. For liquid flows, commonly used dyes are rhodamine B (fluorescein—RhB) and rhodamine 110 (Rh110). Nevertheless, a vast quantity of additional molecular tracers for liquid flows can be found in the literature [164].

The molecular tagging approach is restricted to the induced photoluminescence of molecules and not particles, thus making this flow visualization technique the molecular counterpart of particle image velocimetry (PIV). Because of the substantial difference in the nature of the tracer, MT does not have the complications of the particle-based techniques, such as flow perturbation and poor buoyancy of the tracer particles in confined gas flows at low Reynolds numbers [165]. For this reason, it is currently the most promising technique for flow visualization in microdevices. It is also possible to refer to this technique as laser induced fluorescence (LIF) or phosphorescence (LIP). However, MTT has been adopted here as a more general term, since it comprises both LIF and LIP techniques, and both signals could be used for thermometry purposes in flows.

#### 7.1.1. Photoluminescence: Fluorescence and Phosphorescence

**Figure 30:**
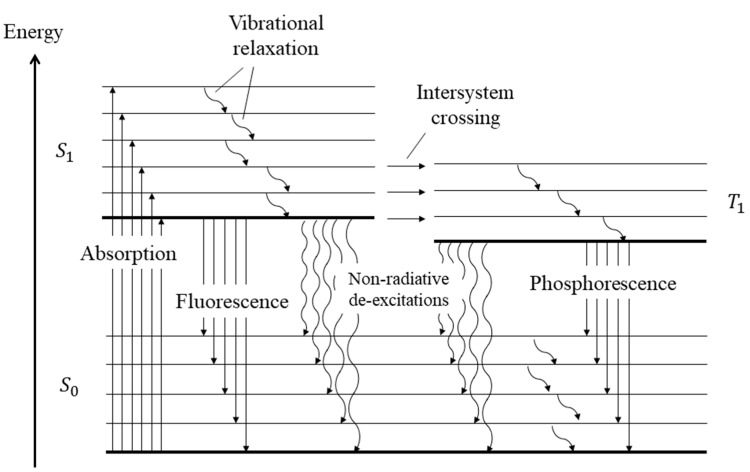
Jablonski energy-level diagram. The straight lines represent radiative processes, the wavy lines represent non-radiative processes. The non-radiative de-excitations include both internal and external conversions [166]. Reproduced with permission of the author.

In the photoluminescence process, the photon absorption at the molecular level necessarily involves an electronic transition from a ground energetic state $S_{0}$ to a higher energetic state $S_{1}$ (molecular excitation). More precisely, an electron of the most external orbital jumps to a higher energetic orbital. A molecule in its excited state is a metastable system since it tends to, after some time, de-excite back to its stable electronic configuration, namely the singlet ground state. The transition of an excited molecule to the ground state can happen through various intramolecular radiative or non-radiative processes. When the de-excitation takes place by means of a radiative process, the molecule loses energy by emitting a photon (see Figure 30).

Two different spontaneous radiative de-excitations to the ground state $S_{0}$ are possible, that is from a singlet $S_{1}$ or triplet $T_{1}$ molecular state. The radiative de-excitation $S_{1}\to S_{0}$ is called fluorescence, while the radiative transition $T_{1}\to S_{0}$ is called phosphorescence. Because of the different nature of the electronic configuration between the $S_{1}$ et $T_{1}$ molecular states, the radiative decay of a singlet molecule has very different characteristics in terms of lifetime and probability with respect to those of the radiative decay of a triplet molecule. Fluorescence happens immediately after the initial molecular excitation and it lasts some nanoseconds (short and intense photon emission phenomenon), while phosphorescence lasts longer and has a higher lifetime, which can vary from a hundred of microseconds to a few milliseconds. A key element to differentiate the temporal mechanisms acting in the phosphorescence phenomenon in respect to the fluorescent phenomenon is the inter-system crossing (ISC), which produces the transition $S_{1}\to T_{1}$ from a singlet to triplet state and is essential for the formation process of phosphorescent molecules.

It should be noted that only a small fraction of excited molecules come back to the ground state through radiative transition, i.e., by emitting luminescence, either fluorescence or phosphorescence. Non-radiative molecular de-excitation is more probable due to phenomena such as inter-molecular collisions, molecular dissociation, and molecular quenching.

The fluorescence quantum yield $\left(\phi_{f}\right)$, that is, the percentage of excited singlet molecules that produce fluorescence, is very low. The main non-radiative transition that limits the fluorescence quantum yield is the inter-system crossing, which transforms a singlet into a triplet. However, even if just a small number of excited singlets radiatively decay to the ground state, the fluorescence intensity is usually stronger with respect to the phosphorescence emission because of the relatively high rate of emission ($k_{f}),$ which is inversely proportional to the characteristic lifetime of the phenomenon $\left(\tau_{f}=1/k_{f}\right)$.

Acetone vapor is characterized by a characteristic lifetime of fluorescence $\tau_{f}$ around 1 µs, but its low quantum yield $\phi_{f}=0.2\%$ reduces the observed fluorescence lifetime to only 4 ns [167]. In diacetyl vapor, the characteristic time of fluorescence emission is of the order of 10 µs (one order of magnitude higher than acetone). Thus, even though its fluorescence quantum yield has the same order of magnitude as acetone, the diacetyl fluorescence intensity is weaker than acetone fluorescence. It is important to notice that some molecules can exhibit only phosphorescent emission (zero fluorescent emission) since their non-radiative transitions are much more probable than their radiative emission from the singlet state. This is the case of benzophenone, in which the energy separation between the lowest energy level in the excited singlet state $S_{1}$ and the lowest energy level in the triplet state $T_{1}$ is so small that the transition $S_{1}\to T_{1}$ is more likely to occur [168].

Phosphorescence is the radiative transition of the triplet molecules to the ground singlet state. Because the internal non-radiative transitions happening in the triplet state are generally slower than the non-radiative transitions happening in the singlet state, the phosphorescence quantum yield is between one to two orders of magnitude higher than the fluorescence quantum yield. The characteristic time of phosphorescence $\tau_{ph}$ is about 10 ms for both acetone [169] and diacetyl [170]. However, the observed lifetime of the phosphorescence emission is about 200 µs for acetone and 1.5 ms for diacetyl [171]. This is due to the fact that the internal non-radiative rate of the diacetyl triplet state is one order of magnitude slower than that of the acetone triplet and, therefore, the phosphorescence quantum yield of diacetyl is $\phi_{ph-dia}=15\%$, which is one order of magnitude higher than that of acetone, which is $\phi_{ph-ace}=1.8\%$. These values are representative of the competition between radiative and internal non-radiative transitions. However, in the presence of external conversions, i.e., molecular quenching, the observed quantum yield is lower and the emission lifetime is shorter.

**Figure 31:**
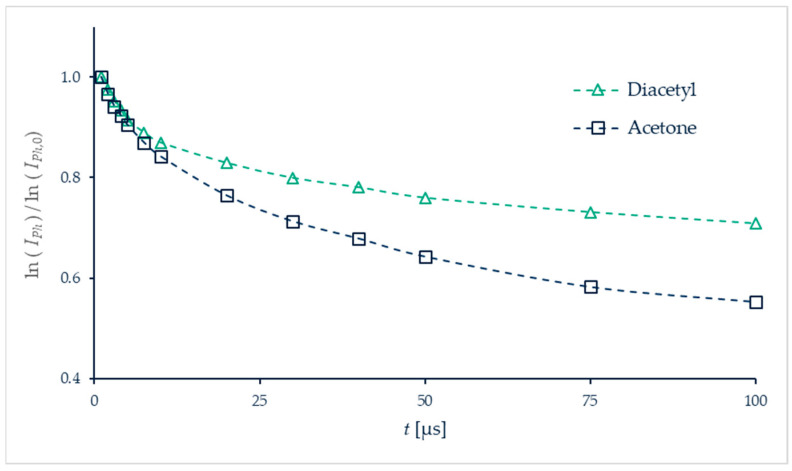
Absolute intensity $I_{ph}$ of phosphorescence emission vs time *t* for vapor acetone (□) excited at wavelength λ=310 nm, and vapor diacetyl (Δ) excited at wavelength λ=410 nm, both at p=5 kPa and T=293 K, and normalized by the intensity value $I_{ph,0}$ at t=1 μs. Data from Fratantonio et al. [172].

Recently, the different behavior of the two tracers were measured at low pressures by Fratantonio et al. 2018 [172]. Figure 31 shows the normalized signal decay with time for both acetone and diacetyl phosphorescence. The diacetyl signal tends to last longer than the acetone signal.

#### 7.1.2. Photoluminescent Properties of MT Tracers

Fluorescence and phosphorescence can be laser induced. The excitation wavelength of the light source is to be determined based on the absorption cross-section and the fluorescence quantum yield, while the quantum efficiency of the photodetector should be compatible with the emission spectrum of the employed tracer. As described by Thurber et al. [173], the signal obtained from a fluorescence emission depends on several parameters

(8) $$I_{f,ph}=\eta_{opt}\frac{E}{hc/\lambda }dV_{c}n_{abs(T)}\sigma_{f,ph(\lambda ,T)}\phi_{f,ph(\lambda ,T)},$$

where $\eta_{opt}$ is the overall efficiency of the collection optics, $E\left[{J\;m}^{-2}\right]$ is the laser fluence, $hc/\lambda \left[{J\;m}^{-2}\right]$ is the energy of a photon at excitation wavelength λ[m], and $dV_{c}\left[m^{3}\right]$ is the collection volume. The temperature-dependent quantities are the number density $n_{abs}\left[m^{-3}\right]$ of absorbing molecules, the molecular absorption cross-section $\sigma \left[m^{2}\right]$ of the tracer, and the quantum yield ϕ. Note that both σ and ϕ depend on the excitation wavelength λ as well as on the temperature *T*. In order to experimentally determine these temperature-dependent properties, it is important to control all the sources of imprecision that might contribute to the wrong estimation of the remaining parameters in the equation. Equation (8) can also be extended to phosphorescence emission, and the subscripts *f* and *ph* represent fluorescence and phosphorescence, respectively.

**Figure 32:**
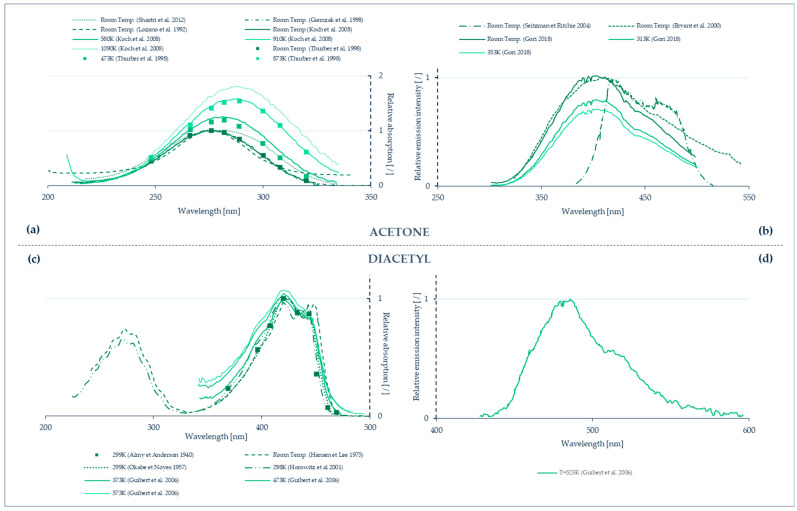
(**a**) Absorption spectra of acetone at different temperatures, from Thurber et al. [173]; (**b**) Fluorescence emission spectra of acetone, from Tran et al. [174]; (**c**) Absorption spectra of diacetyl, from Stier et al. [176]. (**d**) Fluorescence emission spectra of diacetyl, from McKenzie et al. [177]. All the measurements obtained at room temperature have been normalized by their peak value. Measurements done at other temperatures have been normalized with respect to the corresponding room temperature peak value, except the absorption and emission measurements from Guibert et al. [181], which were normalized by the peak values at 373 and 523 K, respectively. Data from [173,174,176,177].

Acetone absorbs in the range 225–340 nm with a maximum absorption cross-section at about 275 nm at room temperature [173] (Figure 32a). A photoluminescence emission prevails in the visible spectrum ranging from 350 to 550 nm for fluorescence and has a wider range for phosphorescence. To our knowledge, only Tran et al. [174] have measured the spectral emission of acetone phosphorescence (Figure 32b), but only at its liquid state. The fluorescence spectra of acetone vapor have been provided by Bryant et al. [175] and Lozano et al. [171]. The measured spectral emission of vapor acetone fluorescence is quite similar to the spectral emission of liquid acetone shown in Figure 32c.

Diacetyl vapor absorbs in the 225–320 nm and 350–470 nm ranges with a maximum absorption cross-section at about 420 nm at room temperature [176] (Figure 32c). The photoluminescence emission prevails in the visible spectrum, ranging from 440 to 500 nm for fluorescence and from 500 to 600 nm for phosphorescence, regardless of the excitation wavelength employed [177] (Figure 32d). Similar results on the phosphorescence spectrum of diacetyl vapor can be found in [178]. Parmenter and Poland [179] and Okabe et al. [180] reported that the fluorescence emission peak happens at 460 nm, and the phosphorescence emission peak occurs at 510 nm, close to the value shown in Figure 32d.

**Figure 33:**
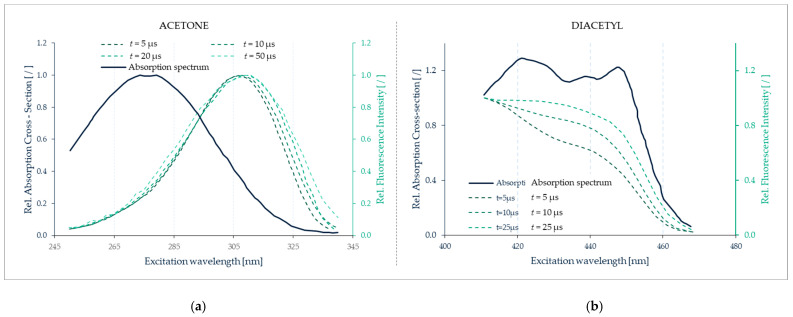
Absorption (solid black lines) of (**a**) acetone, obtained by Lozano et al. [171], and (**b**) of diacetyl, obtained by Stier and Koochesfahani [176] and phosphorescence emission (dashed green lines) intensity of (**a**) acetone, and (**b**) diacetyl, as a function of the laser excitation wavelength, obtained by Fratantonio et al. [172]. The signals are normalized by the intensity value for the wavelength of peak emission. (**a**) Acetone vapor at p=15 kPa and T=293 K; (**b**) Diacetyl vapor at p=5 kPa and T=293 K. Data from [172].

However, as reported by Fratantonio et al. [172], the peak of the absorption wavelength for acetone and diacetyl does not necessarily coincide with the optimal wavelength that needs to be used to excite the molecules for obtaining the highest phosphorescence emission intensity (Figure 33). In the case of acetone, the phosphorescence emission peak is found for an excitation wavelength of around 310 nm, while the absorption peak is at around 275 nm. In the case of diacetyl, even if the whole spectrum of possible excitation wavelengths was not tested, one can readily see that the maximum absorption wavelength at around 420 nm does not coincide with the maximum light emission intensity. The obvious conclusion is that the choice of the excitation wavelength has to be optimized as a function of the chosen molecule and the target application, such as thermometry or velocimetry or both together, for specific gas concentrations.

**Figure 34:**
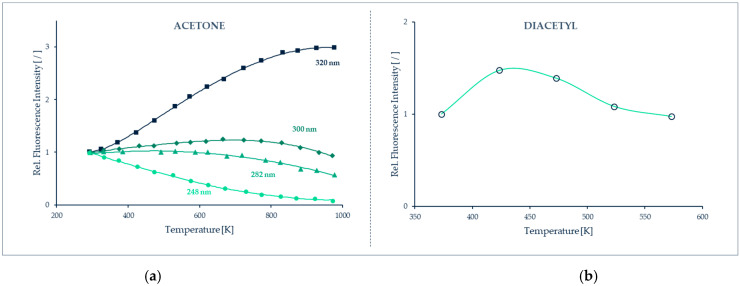
(**a**) Fluorescence emission signal per unit laser fluence of acetone vapor as a function of temperature for different laser excitation wavelengths, obtained by Thurber et al. [173]. The values are normalized by the emission signal obtained at room-temperature. (**b**) Fluorescence emission signal of diacetyl vapor in nitrogen as a function of temperature at a pressure of 0.1 MPa, obtained by Guibert et al. [181]. Data from [173,181].

Temperature dependence of fluorescence and phosphorescence: as reported by Thurber et al. [173] and Guibert et al. [181], the fluorescence emissions intensities of acetone and diacetyl vapors vary with temperature. The knowledge of emission intensity variation with temperature is crucial in order to correlate signal gradients to temperature gradients in the flow field. However, the reported emission intensity variations have been measured for very large temperature variations with respect to the ambient temperature. Thurber et al. [173] thoroughly characterized acetone fluorescence in a temperature range of 295–1000 K at several laser excitation wavelengths ranging from 248 to 320 nm (Figure 34a). Guibert et al. [181] only used a single wavelength at 355 nm for diacetyl in a temperature range of 373–573 K (Figure 34b). It is to be noticed that the results of [173] and [181] were obtained for a flow streaming in a visualization cell at constant pressure: by changing the temperature of the gas flow, the density of the gas mixture was affected, too, and thus no control on the number density of the molecular tracer was provided in these results. However, it is possible to clearly notice the influence of temperature with respect to the obtained signal.

In the literature, there is an evident lack of precise data of luminescent signal emission as a function of temperature in the 300–400 K range for low gas concentrations, which are to be considered the typical thermodynamic conditions of gas in microfluidics applications.

Moreover, it is to be noticed that almost no information exists in the literature for phosphorescence vapor emission dependence on temperature both for acetone and diacetyl. These data would be of great importance for combined molecular tagging velocimetry (where a delay is necessary between tagging and luminescence of the molecules in order to measure their displacement) and thermometry applications.

**Figure 35:**
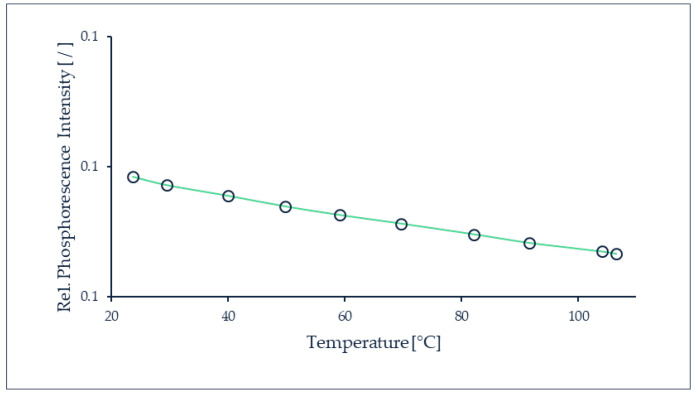
Temperature dependence of diacetyl phosphorescence, measured by Jian-Bang et al. [182]. The data are relative to initial phosphorescence, obtained from measurements at t=20 μs (1% of the phosphorescence lifetime) and extrapolated at t=0. Data from [182].

To the best of our knowledge, one of the few works related to temperature dependence of a phosphorescent signal was realized by Jiang-Bang et al., who investigated the phosphorescence emissions of diacetyl mixed with nitrogen in a static cell by means of a laser monochromatic source at 425 nm [182]. As can readily be seen in Figure 35, the phosphorescence emission was weakly dependent on the variation of temperature, and only a linear decrease of 5% was observed in the temperature region of 20–100 °C.

**Figure 36:**
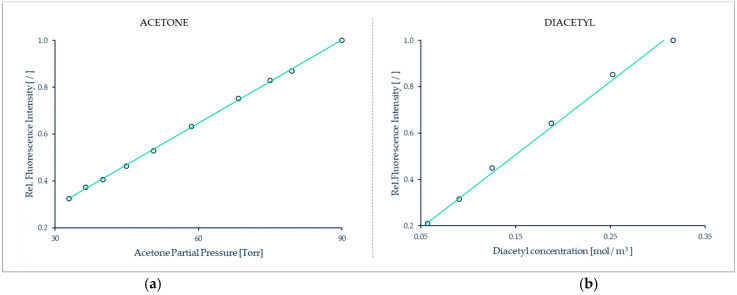
(**a**) Acetone fluorescence emission dependence on partial pressure, according to the data from Lozano et al. [171]; (**b**) Diacetyl fluorescence emission dependence on concentration (at constant temperature, this parameter is easily linked to pressure dependence), according to the data from Tomita et al. [183].

Pressure dependence of fluorescence and phosphorescence: as reported by Lozano et al. [171], the fluorescence emission of acetone vapor has a linear dependence with its partial pressure for constant gas temperature (Figure 36a). As reported by Tomita et al. [183] for the case of diacetyl vapor used as tracer in an air-hydrogen mixture, the intensity of fluorescence emission has a linear dependence to the molecular concentration per unit volume which is directly proportional to partial pressure (Figure 36b).

**Figure 37:**
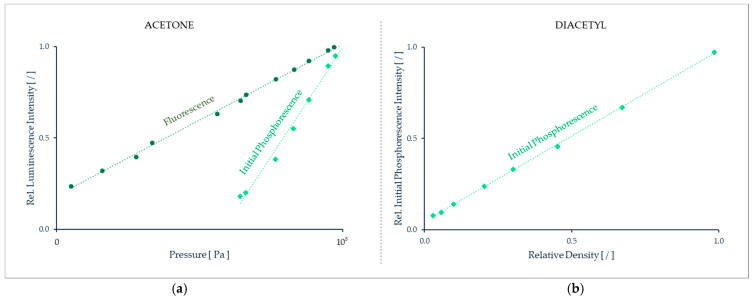
(**a**) (◆) Pressure dependence of acetone phosphorescence emission intensity collected for 100 ns < *t* < 600 ns, normalized by its value at atmospheric pressure for T=294 K and a molar fraction of acetone of 10.4%; (●) experimental fluorescence intensity collected for 0 < t < 10 ns normalized by its value at atmospheric pressure. Data from Si Hadj Mohand et al. [184]. (**b**) Density dependence of initial phosphorescence intensity of diacetyl: the ordinate is normalized by the largest initial phosphorescence intensity of each experimental run, and the abscissa is normalized by the highest density of each experimental run. Data from Jian-Bang et al. [182].

Phosphorescence emission intensity of both acetone [184] and diacetyl [182] is also dependent on the molecular concentration per unit volume, or on the partial pressure of the tracer in the background gas, as shown in Figure 37a,b.

### 7.2. Applications of MTT in Gas Flows at the Macroscale

**Figure 38:**
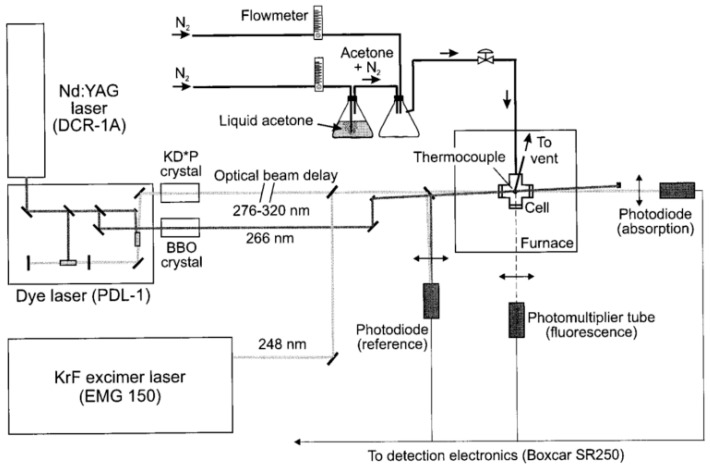
Experimental schematic from Thurber et al. [173] for acetone fluorescence and absorption temperature-dependence experiments. Reprinted by permission from Ref. [173], Applied Optics, Copyright © 2022 The Optical Society.

Early efforts to measure gas temperature at the macroscopic scale by means of laser induced fluorescence were realized in visualization chambers with gas under static or stationary flow conditions. A typical experimental setup, such as the one represented in Figure 38, allows the control of gas thermodynamic conditions within the visualization chamber. This installation, developed by Thurber et al. [173], is composed of a test cell or visualization chamber, with at least two optical accesses; one is transparent to the laser source wavelength and allows molecular tagging, and the other is transparent to visible or UV light, for the fluorescence or phosphorescence emission acquisition. Pressure and temperature of the gas mixture can be regulated in the visualization chamber. The concentration of the gas tracer inside the gas mixture can be controlled by means of flowmeters disposed before the inlet of the test cell. A set of monochromatic lasers that provide light beams at different wavelengths are needed in order to tag the molecules at different excitation frequencies; nevertheless, for some applications, only one monochromatic laser is used. The acquisition system is composed of a photomultiplier tube that can detect the fluorescence emission and a couple of photodiodes that can measure the intensity of the laser beam at the inlet and outlet of the cell, in order to (i) obtain a laser energy measurement that can be used to normalize the fluorescence emission signal, and (ii) measure the absorbance coefficients of acetone in the test cell. Recent measurement techniques use charge-coupled devices (CCD) in order to acquire an image of the emitted fluorescent or phosphorescent signal for flow visualization [172,185].

**Figure 39:**
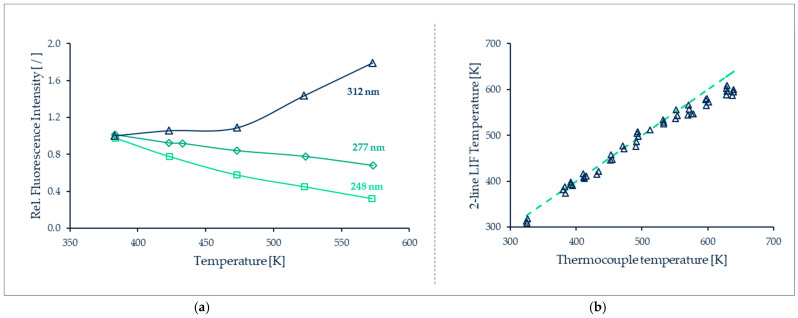
(**a**) Temperature dependence of the relative LIF intensity $I_{f,\lambda }$ of 3-pentanone, normalized with respect to LIF intensity at 383 K; (**b**) Temperature measurement using the dual-wavelength fluorescence (or two-line LIF) technique with $\lambda_{2}=312\;\mathrm{nm}$ and $\lambda_{1}=248\;\mathrm{nm}$, compared to measurements provided by a thermocouple. Data from Grossmann et al. [186]. Data from [186].

Grossmann et al. characterized the LIF dependence on temperature in the 350–640 K range of 3-pentanone. All measurements were realized with a constant number of molecules per unit volume at atmospheric pressure [186]. A dual-wavelength technique was used: for two different excitation wavelengths, $\lambda_{1}$ and $\lambda_{2}$, the fluorescence intensity was measured as a function of temperature and the data were normalized with respect to the signal intensity at 383 K. The normalized intensity ratio

(9) $$\frac{I_{f,}{}_{\lambda_{2}}\left(T\right)}{I_{f,\lambda_{1}}\left(T\right)}=\frac{\sigma \left(\lambda_{2},T\right)\phi \left(\lambda_{2},T\right)}{\sigma \left(\lambda_{1},T\right)\phi \left(\lambda_{1},T\right)}$$

was then calculated and correlated to the gas temperature (Figure 39a). This ratio provides a temperature dependent quantity only, since the number density cancels out (see Equation (8)). The LIF results were in good agreement with thermocouple readings (Figure 39b).

**Figure 40:**
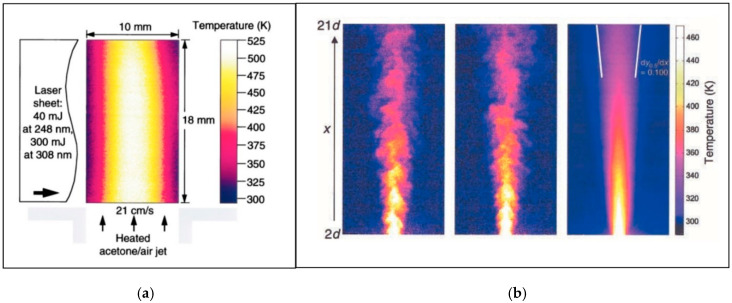
(**a**) PLIF Thermometry measurements from Thurber et al. [187] of a laminar jet heated at 515 K. Reprinted by permission from Ref. [187], Optics Letters, Copyright © 2022 Optical Society of America; (**b**) PLIF Thermometry measurements from Thurber and Hanson [185] of a turbulent jet heated at 465 K. Left and center are instantaneous images. Right is a 100-frame averaged image. Reprinted by permission from Ref. [185], Experiments in Fluids, Copyright © 2022 Springer-Verlag.

Thurber et al. [187] applied the dual-wavelength technique in a measurement of the temperature field of a laminar jet of air seeded with 20% of acetone at atmospheric pressure. The employed excitation wavelengths were 308 and 248 nm. The jet, issued from a 10 mm nozzle at a Reynolds number Re=60, was heated to 515 K on its center line. Planar laser-induced fluorescence (PLIF) was implemented in order to tag the tracer molecules in the plane illuminated by the laser sheet. The two images obtained from the two excitations with different wavelengths were collected by the same CCD. The experiments revealed temperature variations in the flow from 300 to 525 K. The measurement uncertainty was estimated to be 0.6% (Figure 40a). Later on, Thurber and Hanson [185] applied the same technique to a turbulent jet at a Reynolds number Re=5100, with very good results. The air at atmospheric pressure was seeded with 9% acetone, and the flow issuing a 1.7 mm diameter nozzle was heated to 465 K. Important information on the spatial resolution of the measurement was given: a region of the flow of 33 × 42 mm^2^ was investigated and the CCD allowed a spatial resolution of 6.7 µm per pixel. However, in order to improve the signal to noise ratio, a 2 × 2 binning procedure was used (Figure 40b). The temporal resolution was 500 ns. The uncertainties reported on the temperature measurement gradients were the same as in their previous work, but with a slight offset of 10 K in the absolute values. This offset was attributed to the non-homogenous seeding tracer concentrations from the inner to the outer flows. It is to be noticed that this setup allowed the performing of parallel measurements of temperature and tracer molar fraction. In this case, the techniques are greatly complexified [185].

Bryant et al. [175] undertook an LIF study on low temperatures, from 240 K to room temperature, at an excitation wavelength of 266 nm. This work extends the temperature range investigated by Thurber et al., showing a weak dependence of the acetone fluorescent signal on temperature, corresponding to 8% of difference between room temperature and 240 K. It is to be noticed that this experiment was performed in a static cell filled with gas, and thus it was easier to control the concentration of the acetone tracer molecules.

Bessler et al. [188] investigated temperatures in Bunsen-type laminar flames at atmospheric pressure by PLIF. They were able to measure temperatures in a range of 250–2250 K with nitrogen-oxide (NO) as a tracer, using a 248 nm KrF excimer laser that was Raman shifted to its first anti-Stokes beam (~226 nm). The obtained images had a nominal window area of 115 × 80 pixels, each pixel corresponding to a real image area of 260 × 260 µm^2^. LIF signals of 10 laser pulses (20 Hz pulse rate) were integrated on the CCD chip to enhance the signal over noise ratio and to reduce the influence of pulse-to-pulse fluctuations in the laser energy. Between the data sets, temperature measurements of the peak flame values around 2250 K varied about 160 K. Thus, even if the authors considered the precision of the experiments be very good (1.6% for one data set), and the overall accuracy was in the order of 8%. Temperatures and density investigation in flames by PLIF with a hydroxyl radical (OH) as a tracer was also performed by Schiessl et al. [189] and Kostka et al. [190].

Petterson et al. [191,192] have achieved PLIF temperature measurements in confined complex geometries for combustion applications in spark-ignition engines, by using toluene as a tracer. These measurements were coupled with particle image velocimetry. They had to be realized by synchronizing the laser shots and image acquisition with respect to the periodic oscillation of the crank angle of the piston, thus demonstrating that the PLIF technique might be suitable also for unsteady but periodic problems. The viewing plane area was 25 × 30 mm^2^ with a spatial resolution of 0.08 × 0.08 mm^2^/pixel. The uncertainty of the measurement varied from ±5 K at 295 K to ±29 K at 550 K.

**Figure 41:**
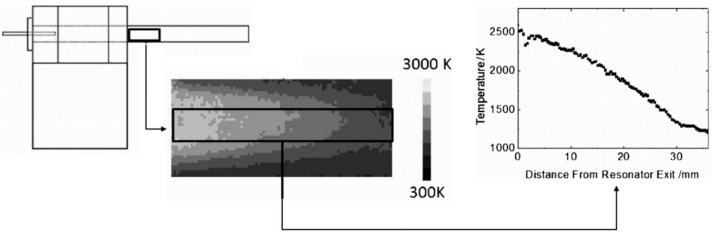
Temperature measurement by Hecht et al. [193] in a confined plasma flow of oxygen, nitrogen and NO as tracer. Left: measurement location; Center: two-dimensional temperature distribution; Right: temperature profile along the axis, averaged over the central 10 mm, limited by the black rectangle in the center image). Reprinted and adapted by permission from Ref. [193], Zeitschrift für Physikalische Chemie, Copyright © 2022 Oldenbourg Wissenschaftsverlag.

Hecht et al. [193] realized an interesting study on temperature fields of a confined plasma flow at low pressure (2–10 kPa) in a quartz tube 25 mm in diameter. The temperature range investigated was quite large, from 300 to 3000 K, and the measurements were obtained with a precision of ±2% (Figure 41). The gas was a mixture of oxygen, nitrogen, or argon in some cases, with a small percentage of tracer (NO). A dye laser pumped with the second harmonic of a Nd:YAG laser was used to generate light at a wavelength of 615 nm, which was sum frequency-mixed with the third harmonic of the Nd:YAG laser to produce a beam in the wavelength region around 225.1–225.2 nm, with a laser pulse energy of 10 mJ, enabling NO excitation. The use of a band of different wavelengths was essential in order to compare the signal emission response to different temperatures. The investigated region had a of 36 × 20 mm^2^ area, but no information on the pixel spatial resolution was given.

Another example of PLIF applied to confined spaces was provided by Notthoff et al. [194], who investigated the time temperature profile of the gas phase in the chemical vapor synthesis process of fabricating nanoparticles. The investigated geometry corresponded to a thin wall alumina (Al_2_O_3_) ceramic tube with several optical accesses for the laser sheet entrance and acquisition purposes. The field of view for each access corresponded to an area of approximately 5 × 15 mm^2^. No details were provided on the spatial resolution of the measurements. The temperature measurements were realized in the 500–1100 K range at 2 kPa. No uncertainty values were given for the measured temperatures. The tracer gas, NO, was seeded in the carrier gas, helium with and without 2-propanol.

To the best of our knowledge, even if the laser induced fluorescence technique has been widely used for thermometry applications in the gas phase at the macroscale, it has yet to be used for gas flows at the microscale. Furthermore, the phosphorescent emission of tracer molecules at the vapor phase has not been employed in the literature for thermography purposes in macroscopic nor in microscopic gas flows.

### 7.3. Applications of MTT in Liquid Micro-Flows

Fogg et al. [195] measured the void fraction and the liquid temperature of air-water flow in microchannels by LIF. The experiments were carried out in a microchannel of 500 µm width, 100 µm depth, and 2.5 cm length. The microchannel was fabricated by deep reactive ion etching (DRIE) in a silicon substrate, and sealed by a 500 µm thick glass wafer. The ratio of fluorescent emissions of Rhodamine B (RhB), which is highly temperature dependent, and Rhodamine 110 (Rh110), which is poorly temperature dependent, was used to generate the calibration curves in a temperature range of 40 to 100 °C. This approach was able to obtain local liquid temperatures within an uncertainty of ±3 K. For the signal acquisition, a photodiode coupled to a microscope was used instead of a CCD. An interrogation window of 1.3 × 0.5 mm^2^ was used and the fluorescent signal for temperature measurements was integrated into this area. Thus, the interrogation window corresponded to the spatial resolution of the measurement. The error in the temperature measurement became excessive as void fraction increased, making measurements possible for instantaneous void fraction below 0.05.

Motosuke et al. [196] developed a high temporal and spatial resolution LIF experimental system. Temperature measurements were realized in a rectangular cross-section microchannel made of borosilicate glass (with 500 μm width, 50 μm depth and 50 mm length). A mercury lamp was used in order to excite the fluorescent-dye (fluorescein) at its peak excitation wavelength of 494 nm, while a monochromic laser with a 635 nm wavelength was used to heat the Brilliant Blue FCF (BB) non-fluorescent-dye in water. The specific choice of fluorescein as temperature dependent dye for this study was essential since it does not absorb in the wavelength of the heating laser. Calibration curves of fluorescent signal intensity as a function of temperature were established for different BB concentrations. The spatial resolution was given by the Rayleigh limit and was estimated to be 530 nm. The temporal resolution was dictated by the high-speed camera employed, the fastest acquisition time of which was 500 µs. In order to increase the signal over noise ratio, the signal was phase-averaged over several images. Via this technique, fast temperature variations with time were measured in the 283 to 343 K range with a ±0.5 K uncertainty.

**Figure 42:**
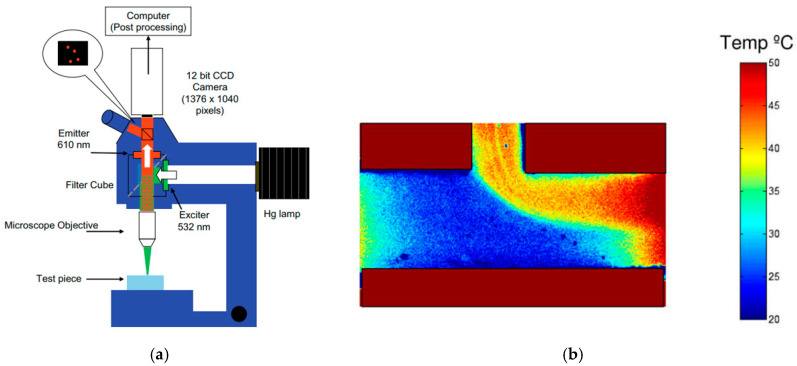
(**a**) µLIF thermometry experimental setup from Chamarthy et al. [197]; (**b**) temperature measurements in a T-junction. Reprinted by permission from Ref. [197], International Journal of Heat and Mass Transfer, Copyright © 2022 Elsevier Ltd.

Chamarthy et al. [197] measured the temperature in a ‘T’ junction at the mixing plane of hot and cold deionized water. The experimental setup used corresponded to a standard µPIV setup adapted for µLIF purposes (Figure 42). The channel was 500 µm wide and 200 µm deep. The authors applied the dual-wavelength measurement principle by using RhB and Rh110 as tracer molecules. Temperature gradients in the liquid mixture were measured with an uncertainty of ±1.1 to ±2.7 K for single-pixel measurements in the temperature range of 20 to 50 °C. The spatial resolution of the measurement was not clearly specified.

Kim et al. [198] through the dual-tracer fluorescence thermometry (DFT) technique, also known as the ratiometric approach, carried out temperature measurements of water in the range of 20 to 60 °C for a laminar Poiseuille flow through a 28 mm long PDMS-glass channel heated on one side and with a square cross section 1 mm wide. The novelty of this work consisted in coupling Fluorescein (FI), a temperature sensitive tracer whose fluorescent intensity is directly proportional to temperature, and Sulforhodamine B (SrB), whose fluorescent intensity is inversely proportional to temperature. This clever approach allows for the increasing of the sensitivity of the DFT approach. The choice of these tracers is also particularly appropriate since both FI and SrB can be excited at 514 nm, while their emission spectra have little overlap (Fl peak at 518 nm and SrB peak at 591 nm). Furthermore, contrarily to RhB, the FI molecule has negligible absorption on fused-silica surfaces at 20–60 °C. The authors observed a temperature sensitivity of 4 to 12% per K. The uncertainty in temperature measurements was estimated to be about 0.3 K for a spatial resolution of 30 µm. This technique could not measure temperatures in transient phenomena, nevertheless it could be adapted for this purpose by using a set of two cameras and appropriate filters as demonstrated by Shafii et al. [199] and Natrajan et al. [200] for macroscopic flows.

Recently, Park et al. [201] have measured temperatures of supercritical carbon dioxide (CO_2_) in a microchannel with a hydraulic diameter of 300 µm and a length of 12 mm. The temperatures ranged from 23 to 90 °C at a nominal pressure of 79 MPa. The temperature of the flow was regulated by an external electrical heater. The flow transited from liquid to supercritical phase for temperatures above 31 °C. A single-dye single-color method was used with a single excitation at 532 nm. The chosen tracer was rhodamine 6G, which has poor solvability in CO_2_ but a great quantum efficiency; therefore, little quantities are needed in order to obtain a temperature sensitive fluorescent signal. The images were collected by coupling a microscope to a camera with CCD resolution of 768 by 768 pixels and a spatial conversion factor of 4.2 µm per pixel. The exposure time was 41 ms with a repetition rate of 24 fps. The laser beam affected the totality of the fluidic plane. Nevertheless, no temperature gradient was obtained, but the registered temperature varied with time. From the measurements, one could clearly see the temperature dependence of the fluorescence signal generated by the tracer in time. The uncertainty of the measurement was quite high (±10 K for some cases).

An interesting heat transfer problem in a minichannel with a complex zig-zag geometry and a 4 × 4 mm^2^ cross section was studied by Shi et al. [202]. The temperature gradients arising along the length of the channel were clearly revealed by two-color two-dye PLIF experiments. The laser sheet plane (500 µm thick and 12 cm wide) could be adjusted inside the channel in order to obtain tagged molecules in a specific plane only. One pulsed monochromic Nd:YAG laser doubled at 532 nm was used. Two CCDs coupled with appropriate filters were used for the signal acquisition of the emissions of FI (543–549 nm) and SrB (>633 nm). This choice of acquisition wavelengths was adopted in order to obtain signal intensities of the same order of magnitude in the two CCDs. Through this method, temperature gradients along the three coordinates of space were obtained. The uncertainty on the absolute temperature measurement was lower than ±3% in the 17–60 °C range. The spatial resolution corresponded to approximately 9.4 µm, with a CCD of 1376 × 1040 pixels for a 13 × 7 mm^2^ field of view.

### 7.4. Perspectives for MTT in Gas Micro-Flows

Until today, no molecular tagging thermography measurements have been realized in micro gas flows, and as it can be readily seen, much work is needed in order to be able to use the technique for this application. Nevertheless, MTT can be considered as an excellent candidate for temperature field measurements of gas flows in micrometric confined environments, mainly due to the low intrusiveness of the technique.

Encouraging points to be noticed are that the CCD and the intensification relay optics technologies are making great leaps in efficiency, and it has thus become possible to image low intensity luminescent signals such as the ones to be found in gas microflows, with increasing sensitivity [172]. This technological advancement might also improve the temporal resolution of the technique by enabling one-shot-one-image acquisitions and thus lowering the signal acquisition time. Therefore, the lower acquisition time limit can be defined by the characteristic lifetime of the fluorescence (a few ns) or phosphorescence emission (a few ms). Furthermore, previous molecular tagging velocimetry works have demonstrated that enough spatial resolution can be achieved when measuring gradients of luminescent signals at the microscale in gases [162]. Another important point to be added is that the main technological barriers with respect to manufacturing techniques for obtaining leak tight fluidic chips for gas applications are being bypassed on a day-to-day basis, thus making possible the fabrication of new interesting devices where thermography measurements can be performed.

Current drawbacks of this technique might be the limited choice of the fluorescent vapor tracers to be used, which are often toxic or have poor sensitivity to temperature changes.

**Figure 43:**
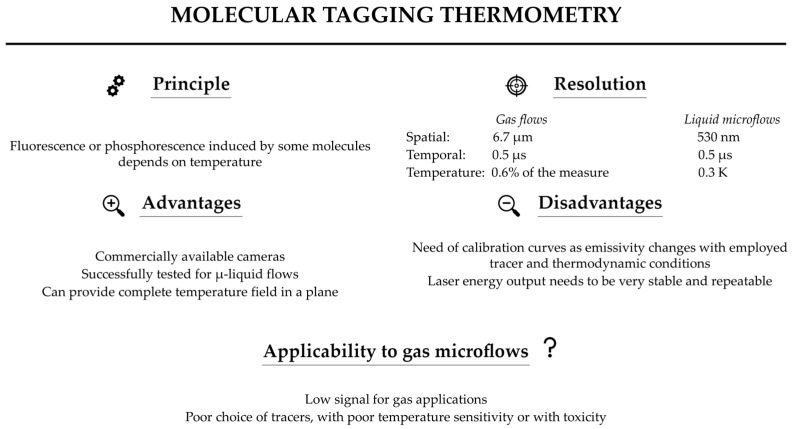
Summary of molecular tagging thermometry features. For gas flows, values of spatial and temporal resolutions are from [185], and value of temperature resolution is from [187]. For liquid flows, values of spatial and temporal resolutions are from [196], and value of temperature resolution is from [198].

Figure 43 summarizes the main features of MTT.

## 8. Conclusions and Perspectives for Thermometry in Gas Microflows

This review has presented the various experimental techniques currently available for thermometry in microflows and classified them according to the exploited physical principle. Following an analysis of the implementation of each technique, its applicability at microscale has been discussed, pointing out its advantages and disadvantages, and providing the orders of magnitude of its spatial, temporal and temperature resolutions. The feasibility and the factors limiting or favoring the implementation of each technique to the thermometry of gaseous microflows have then been highlighted.

Though temperature microsensors based on thermal expansion or thermoelectric effects are relatively simple to implement, their applicability at the microscale is strongly limited, for the following reasons:

- the sensors are invasive and their effect on the flow structure and the temperature distribution is non-negligible at microscale;
- the temperature is obtained only at discrete locations.

For this reason, the review has been focused on optical techniques based on thermo-optic effects. Some of these techniques (IR thermometry, and thermometry with TLCs and TSPs) can provide temperature distributions at the wall, and the other ones can provide temperature distributions within the fluid. The later can be slightly intrusive (thermometry with TLCs and TPPs), low-intrusive (RT and MTT), or non-intrusive (IBT).

Interferometry-based thermometry (IBT) is hardly applicable to gases at the microscale due a too low resolution limited by both the low density of gases and the small dimensions. As a consequence, particular emphasis has been given to Raman thermometry (RT) and molecular tagging thermometry (MTT), which are the most promising techniques for thermometry within the gas and in confined flows. Although their implementation requires quite complex equipment, these two techniques are able to provide local temperature information in rarefied flow conditions with very low intrusiveness. The current limits are linked to the difficulty in obtaining experimental data close to the wall, and some challenging issues have still to be solved before being able to measure temperature jumps at the wall in slip flow regimes.

In future studies, it would be interesting to associate these two techniques in order to benefit from the advantages of both of them and to compare their pros and cons in greater detail. As a next step, they should be coupled to techniques developed for wall temperature measurements, such as TSP thermometry. A final goal would be to simultaneously perform thermometry and velocimetry, which is theoretically possible with molecular tagging techniques.

## Data Availability

All data are taken from the literature and are available in the referenced papers.
